# Exploring the *Tenebrio molitor* gut microbiota response to LDPE and PET: putative genetic indicators and methodological insights

**DOI:** 10.3389/fmicb.2026.1746922

**Published:** 2026-06-15

**Authors:** K. Biełło, G. Rodríguez-Caballero, D. Becerra-Mora, N. Dorado-Blanco, L. P. Sáez-Melero, C. Moreno-Vivián, V. M. Luque-Almagro, A. Olaya-Abril, M. D. Roldán

**Affiliations:** Departamento de Bioquímica y Biología Molecular, Edificio Severo Ochoa, Campus de Rabanales, Universidad de Córdoba, Córdoba, Spain

**Keywords:** functional annotation, insect gut microbiome, plastic biodegradation, shotgun metagenomics, *Tenebrio molitor*

## Abstract

Insect gut microbiomes are recognized as potential reservoirs of enzymatic activities relevant to plastic metabolism. Here, we investigated the taxonomic and functional dynamics of the *Tenebrio molitor* gut microbiota under dietary exposure to low-density polyethylene (LDPE) and polyethylene terephthalate (PET) using 16S rRNA sequencing and shotgun metagenomics. Significant compositional shifts were detected at the ASV level, with plastic-fed cohorts showing enrichment of taxa implicated in xenobiotic metabolism. Predicted functional changes suggested altered abundance of pathways related to aromatic compound processing and redox homeostasis. Metagenomic assembly and functional annotation, performed through a reproducible open-source workflow, revealed several putative proteins with distant homology to enzymes such as phthalate dioxygenases, urethanases, and polyhydroxyalkanoate depolymerases. A metagenome-assembled genome (MAG) assigned to *Enterococcus* accounted for most recovered protein-coding sequences. Although gene-level comparisons did not show statistically significant differences, Gene Set Enrichment Analysis (GSEA) highlighted ABC transporter signatures and stress-response ATPases under plastic-exposed conditions. Overall, this exploratory study reveals microbial shifts and putative genetic indicators of metabolic potential within the *T. molitor* gut, providing a reproducible analytical framework for future investigations into the microbial role in plastic bioconversion.

## 1 Introduction

Plastic pollution represents one of the most persistent and complex environmental threats of the Anthropocene. With global plastic production exceeding 400 million metric tons per year, a substantial fraction—estimated at over 75%—ultimately escapes effective collection and accumulates in terrestrial, freshwater, and marine ecosystems (Geyer et al., 2017; Ivleva et al., 2017). The environmental persistence of plastics, especially polyethylene, polypropylene (PP), and polyethylene terephthalate (PET), leads to their progressive fragmentation into microplastics (MPs) and nanoplastics (NPs), which now pervade nearly all ecological compartments. MPs and NPs have been detected in soil, air, rivers, oceans, polar ice, drinking water, and human tissues, including blood, placenta, and lungs (Cox et al., 2019; Prata et al., 2020). These particles act not only as physical stressors but also as vectors for toxic additives and adsorbed pollutants. Specifically, low-density PE and PET have been associated with adverse outcomes ranging from bioaccumulation and oxidative stress in aquatic fauna to inflammation, endocrine disruption, and gut dysbiosis in mammalian models (Yong et al., 2020). As mounting evidence reveals their potential impacts on biodiversity and human health, MPs and NPs have emerged as contaminants of global concern, prompting urgent calls for scalable mitigation strategies.

Despite major policy and technological efforts, current waste management and recycling infrastructures are insufficient to contain the plastic crisis. Conventional mechanical recycling faces limitations due to polymer cross-contamination, additive complexity, and degradation of material quality over successive cycles (Hopewell et al., 2009). Incineration, while reducing plastic volume, generates hazardous emissions and greenhouse gases; conversely, landfilling perpetuates long-term environmental leakage. Even so-called “biodegradable” plastics often require industrial composting conditions rarely met in natural settings (Kale et al., 2007). Chemical recycling technologies remain largely energy-intensive and underdeveloped at scale. The resilience of polymers like low-density PE and PET to abiotic degradation exacerbates the challenge. Hence, there is a critical need for complementary, biologically driven approaches that can target recalcitrant plastics under environmentally relevant conditions.

Among emerging strategies, microbial biodegradation has shown particular promise. Leveraging the metabolic diversity of microbial communities, researchers are increasingly turning to advanced omics technologies, especially metagenomics, metatranscriptomics, and proteogenomics, to uncover enzymes and pathways capable of plastic depolymerization. Unlike traditional cultivation, which accesses only a small fraction of environmental microbiota, shotgun metagenomics and its transcriptomic or proteomic counterpart enable comprehensive, high-throughput exploration of uncultured microbial dark matter (Jahanshahi et al., 2023; Wu et al., 2025). These techniques can identify candidate genes, transcripts, and proteins involved in plastic degradation across entire microbiomes (Gaytán et al., 2020). Metaproteogenomics, in particular, provides simultaneous insight into both genetic potential and actual enzymatic expression, helping to resolve functional redundancy and improve pathway resolution (Takamiya et al., 2024). In parallel, specialized databases such as PlasticDB, PAZy, and PMBD allow researchers to match newly discovered sequences against known plastic-degrading enzymes, accelerating discovery and functional prediction. These integrative approaches hold immense potential for developing efficient microbial consortia or enzyme cocktails for bioremediation and plastic valorization (Salinas et al., 2023).

Several microbial species and enzymatic classes have already demonstrated the ability to degrade synthetic polymers under controlled conditions. The discovery of PETase from *Ideonella sakaiensis*, capable of hydrolyzing PET into mono(2-hydroxyethyl) terephthalate (MHET), terephthalic acid (TPA), and ethylene glycol, marked a pivotal breakthrough (Yoshida et al., 2016). Other enzymes, including cutinases, laccases, monooxygenases, and alkane hydroxylases, have shown activity against PE, polyurethane, and other polymers (Heris and Amobonye, 2024). Bacteria from genera such as *Pseudomonas*, *Rhodococcus*, and *Bacillus*, as well as fungi including *Aspergillus* and *Penicillium*, have been reported to mineralize or biofragment various plastics. Beyond environmental isolates, recent studies have uncovered promising microbial candidates within the guts of plastic-ingesting organisms. Insect larvae—particularly *Tenebrio molitor* (mealworms)—have garnered attention for their capacity to consume and partially mineralize low-density PE and polystyrene (PS), with degradation products including CO₂ and short-chain hydrocarbons (Yang et al., 2015; Brandon et al., 2021). Analyses of larval gut microbiota have identified shifts in microbial composition and metabolic activity following plastic ingestion, revealing bacterial taxa such as *Citrobacter*, *Serratia*, and *Klebsiella* as recurrent participants in plastic degradation (Lou et al., 2019; Brandon et al., 2021). The gut microbiomes of insects thus represent underexplored, metabolically rich environments for enzyme discovery and microbial synergy.

Crucially, while previous studies have successfully documented the capacity of *T. molitor* larvae to ingest plastics, most investigations have relied heavily on 16S rRNA amplicon sequencing, which offers limited functional resolution and relies on speculative metabolic predictions. High-resolution shotgun metagenomic studies targeting the mealworm gut remain scarce, and the technical bottlenecks inherent to this host-associated model are rarely scrutinized. Specifically, comparative metagenomic frameworks simultaneously evaluating polymers with fundamentally distinct chemical backbones—such as the highly recalcitrant aliphatic chains of low-density PE versus the aromatic ester linkages of PET—are noticeably lacking under identical bioinformatic pipelines. Furthermore, there is a profound lack of methodological transparency in the literature regarding how host DNA contamination attenuates the statistical recovery of functional signals in insect gut metagenomes. Therefore, this study aims to bridge these critical knowledge and methodological gaps. We investigate the microbial response and genetic potential of *T. molitor* gut microbiota under exposure to low-density PE or PET diets using a combined 16S rRNA and shotgun metagenomics approach. Beyond mapping taxonomic and functional shifts through an integrative workflow (incorporating PlasticDB, PAZy, PMBD, InterProScan, and GhostKoala), this work provides a rigorous assessment of the quantitative limitations imposed by host genetic backgrounds. This reproducible strategy aims to contribute realistic insights into the microbial dynamics underpinning insect-mediated plastic degradation while offering essential methodological benchmarks for future biotechnological exploration.

## 2 Materials and methods

### 2.1 Experimental design

The study was performed using *Tenebrio molitor* larvae from the mealworm farm Insectalia in Villamantilla (Madrid, Spain). The mealworm larvae were fed with micronized pulverized low-density polyethylene (LD) or pulverized polyethylene terephthalate (PET), which were supplied by the Plastic Technology Center of AIMPLAS (Valencia, Spain). Each polymer was mixed with natural wheat bran in ratio 1:1, and as the feeding control only wheat bran was used. Prior to the experiment, the larvae were fed wheat bran until they reached an average length of approximately 1 cm and a weight of 10 mg. They were then subjected to a 48-h starvation period to eliminate any potential effects of the previously ingested food. The larvae, with a similar weight, were then divided into three groups (100 larvae each) and placed in new plastic containers with an equal amount of feedstock. The first group was a control (C) set, which was fed with 9 g of wheat bran. The second group was fed with 4.5 g LD mixed with 4.5 g of wheat bran, while the last group was fed with 4.5 g PET mixed with 4.5 g of wheat bran. During the experiment, the temperature was maintained at 25 °C and the humidity at 70%. After a 30-day breeding period, the larvae from all three experimental settings were collected, measured, weighed, and subjected to further analysis. All three treatments were performed and analyzed in replicates.

### 2.2 DNA extraction

DNA was extracted from four biological replicates per condition (C, LD, and PET). Each replicate consisted of the pooled guts from 30 larvae. For this purpose, mealworms were euthanized by placing them on ice, and then sterilized by immersing them in ice-cold 75% ethanol, followed by three rinses with ice-cold sterile 0.85% saline water (Lou et al., 2020). Subsequently, sterile blades and forceps were used to dissect the larvae. The entire guts were pulled and placed into a 1.5 mL centrifuge tube. The collected samples were immediately stored at −80 °C for further analysis. DNA was extracted using the QIAamp PowerFecal Pro DNA Kit (QIAGEN, Germany) following the manufacturer’s instructions. The isolated DNA was further purified using the QIAquick PCR and Gel Cleanup Kit (QIAGEN, Germany) and its concentration was quantified by a ND1000 spectrophotometer (Nanodrop Technologies, Waltham, MA, USA). DNA quantification is shown in Supplementary Tables S1, S2.

### 2.3 16S rRNA gene amplicon sequencing and bioinformatics analysis

#### 2.3.1 Library preparation and sequencing

Metabarcoding library preparation and sequencing were conducted by AllGenetics & Biology SL (Galicia, Spain).^1^ To generate the DNA libraries, the V4 region of the bacterial 16S rRNA gene was amplified. This amplification utilized the 515F-Y (5’ GTGYCAGCMGCCGCGGTAA 3′) and 806R-B (5’ GGACTACNVGGGTWTCTAAT 3′) primers, which included sequencing adapters (Parada et al., 2016; Apprill et al., 2015). The PCR program consisted of an initial denaturation step at 95 °C for 5 min, followed by 25 cycles of 95 °C for 30 s, 46 °C for 45 s, and 72 °C for 45 s, concluding with a final extension step at 72 °C for 7 min. Subsequently, unique barcodes for sample multiplexing were attached during a second PCR amplification. This second reaction used identical conditions to the first but was limited to five cycles with an annealing temperature of 60 °C (Vierna et al., 2017). Both PCR reactions were performed using NZYTaq 2x Green Master Mix and polymerase (NZYTech, Lisbon, Portugal). Pooled samples were then sequenced on the Illumina MiSeq PE300 platform (Illumina Inc., San Diego, CA, USA).

#### 2.3.2 Sequencing data processing

Microbiome bioinformatics analyses were performed using Qiime2 version 2024.2 (Bolyen et al., 2019). Upon receipt of demultiplexed paired-end reads from the sequencing service, Illumina adapters were removed with Cutadapt (Martin, 2011). Sequences were then trimmed based on their per-base quality, as assessed by FastQC (Andrews, 2010). Filtered reads were denoised using the DADA2 software version 1.26 (Callahan et al., 2016) to generate amplicon sequence variants (ASVs) (Supplementary Table S1). The resulting ASVs were taxonomically classified against the SILVA ribosomal RNA database, release 138 SSU (Quast et al., 2013), using a pre-trained classifier for the 515F-806R region, implemented via scikit-learn version 0.24.1 (Bokulich et al., 2018). Demultiplexed raw sequence files have been submitted to the NCBI Sequence Read Archive^2^ and are accessible under BioProject PRJNA1295778.

#### 2.3.3 Statistical analyses of microbial community diversity

Multivariate statistical analyses were conducted in R version 4.5.0 (R Core Team, 2024) (Script 1). Community ecology metrics were calculated using the vegan package (v2.6–6.1) (Oksanen et al., 2024). To account for differences in sequencing depth among samples, raw ASV counts were rarefied to the minimum number of sequences observed (sample C3, 8,316 reads). Rarefaction curves were generated to confirm that the selected depth captured the majority of community richness across all samples.

*α*-diversity was assessed at the ASV level using observed richness, Shannon diversity, and Chao1 richness estimates. Normality and homoscedasticity assumptions were evaluated using the Shapiro–Wilk and Bartlett tests, respectively, prior to applying one-way ANOVA followed by Tukey’s HSD *post hoc* test.

*β*-diversity was evaluated using Bray–Curtis dissimilarities computed from the ASV abundance table, followed by non-metric multidimensional scaling (NMDS) in two dimensions. Differences in overall community composition among diet groups were tested via Permutational Multivariate Analysis of Variance (PERMANOVA, 999 permutations), and post hoc pairwise comparisons were performed using the pairwiseAdonis package (Martínez-Arbizu, 2020). Additionally, hierarchical clustering of samples based on Bray–Curtis distances was conducted using Ward’s minimum variance method (ward. D2).

To identify taxa associated with specific diets, an indicator species analysis (ISA) was performed at the ASV, genus, and family levels using the indicspecies package (v1.7.14) (De Cáceres and Legendre, 2009). The multipatt function was applied with 999 permutations to test the statistical significance of indicator values.

Predicted functional profiles were inferred from the ASV table using PICRUSt2 (Douglas et al., 2020). The pipeline involved multiple steps: sequence placement into a reference phylogeny, hidden-state prediction of gene family abundances (e.g., EC numbers), and pathway-level inference using the MetaCyc database. The output was a table of predicted pathway abundances per sample. To identify significantly differentially abundant pathways between groups, functional predictions were analyzed using STAMP (Statistical Analysis of Metagenomic Profiles, v2.1.3) (Parks et al., 2014). Pairwise comparisons were conducted using *t*-test with Benjamini–Hochberg correction for multiple testing. Pathways with adjusted *p*-values < 0.05 and effect sizes above the software’s recommended threshold were considered statistically significant. Three comparisons were performed: PET vs. C, LD vs. C, and C vs. all other samples (PET + LD).

### 2.4 Metagenomic sequencing and bioinformatics analysis

#### 2.4.1 Metagenomic library preparation and sequencing

Metagenomic sequencing libraries were prepared from the n = 12 total biological replicates (four per condition) using the Illumina DNA Prep library preparation kit (Illumina), strictly adhering to the manufacturer’s instructions. Each library was dual-indexed to enable pooling for sequencing and subsequent demultiplexing. The fragment size distribution of the prepared libraries was assessed using an Agilent 2,100 Bioanalyzer with the Agilent HS DNA Kit. Following library preparation, finished libraries were pooled in equimolar amounts based on quantification results from a Qubit dsDNA HS Assay (Thermo Fisher Scientific). The pooled library was then sequenced on a fraction of an Illumina NovaSeq PE150 flow cell. Raw metagenomic sequencing data (~60 Gb) from *Tenebrio molitor* gut samples are available in the NCBI Sequence Read Archive (SRA) under BioProject PRJNA1307007 (twelve BioSamples, SAMN50801444–SAMN50801455; SRA runs SRR50801444–SRR50801455). The corresponding co-assembled metagenome is deposited in GenBank (CoAssembly, SUB15570984), and the refined metagenome-assembled genomes (MAGs) derived from this assembly are also publicly accessible (SUB15571070).

#### 2.4.2 Quality control and preprocessing of sequencing data

Raw paired-end sequencing data, consisting of forward (R1) and reverse (R2) reads with associated quality scores, were received in FASTQ format. The quality of these raw FASTQ reads was initially assessed using FastQC v2.1.0 (Andrews, 2010) and summarized with MultiQC v1.17 (Ewels et al., 2016). Prior to initiating the main metagenomic bioinformatic pipeline, raw reads were aligned to the *Tenebrio molitor* reference genome icTenMoli1.1 (Mann et al., 2024) using Bowtie2 v2.5.4 (Langmead and Salzberg, 2012), and only the unaligned reads were retained for downstream analyses.

A preliminary exploration of the taxonomic composition of the unaligned data was conducted using Kraken 2 v2.1.3 (Wood et al., 2019) with the pre-built “PlusPFP” database. This database includes reference genome/protein sequences from various taxonomic groups (archaea, bacteria, viral, plasmid, human, protozoa, fungi, plant) and UniVec_Core (a collection of common contaminant sequences). The diversity and relative abundance of the taxonomic hits retrieved by Kraken 2 were graphically represented using the Krona package v2.7.1 (Ondov et al., 2011). Additionally, bacterial reads were specifically extracted using the extract_kraken_reads.py script from KronaTools v2.8.1 (Lu et al., 2022), by specifying their NCBI taxonomy ID as ‘txid2’.

The comprehensive analysis of the metagenomic data was performed using the nf-core/mag pipeline v3.2.1 (Krakau et al., 2022), which is based on the Nextflow workflow manager v24.10.2.5932 (Di Tommaso et al., 2017), for assembly, binning, and taxonomic and functional annotation of metagenomes. Initial filtering steps within this pipeline included the removal of adapter sequences, trimming of low-quality regions (defined by a mean quality score threshold below 30), and exclusion of reads shorter than 80 base pairs (bp) using Fastp v0.23.4 (Chen et al., 2018). Subsequently, quality-trimmed reads were aligned against both the PhiX phage reference genome (a common Illumina sequencing control) and the Ensembl *Homo sapiens* GRCh38 genome using Bowtie2 v2.4.2; only unaligned reads were retained for further processing. The number of reads retained after quality filtering and host/contaminant removal, along with host-associated reads, were summarized (Supplementary Table S2).

#### 2.4.3 Metagenomic assembly and binning

A metagenomic assembly bioinformatic protocol was executed to obtain draft metagenome-assembled genomes (MAGs). This pipeline involved joining quality-filtered reads into larger, continuous sequences known as contigs, which were then assigned to distinct draft genomes, or bins. This assignment process accounted for the possibility that identical sequences might originate from different genomes within the complex metagenomic sample.

For the assembly phase, metagenomes were co-assembled using MEGAHIT v1.2.9 (Li et al., 2015), as implemented in the nf-core/mag-Assembly module. The assembly was performed with a range of *k*-mer sizes (21,29,39,59,79,99,119) and a minimum count parameter of -min-count 2. Quality metrics of the resulting raw assembly were subsequently computed with QUAST 5.0.2 (Gurevich et al., 2013), providing information on the number of contigs and total length (Supplementary Table S3).

The resulting assemblies were subjected to binning using the nf-core/mag-Binning module, which employed two distinct software tools in parallel: MetaBAT2 v2.15 (Kang et al., 2019) and MaxBin2 v2.2.7 (Wu et al., 2016). A contig length threshold of 1,000 bp was set for MaxBin2 and 1,500 bp for MetaBAT2. To generate the highest quality bin sets (MAGs) for each sample, DAS Tool v1.1.6 (Sieber et al., 2018), also implemented within the nf-core/mag-binning module, was utilized. This software estimates bin quality and completeness based on a scoring function (Bin score) derived from the frequency of bacterial or archaeal reference single-copy genes (SCGs). A summary table of DAS Tool parameters for the generated bins was produced (Supplementary Table S4). During the refinement step, DAS Tool combined the results from MetaBAT2 and MaxBin2 to select the optimal version of each bin, considering its bin score, N50 value, and overall bin size. This refinement process reduced the number of final high-quality bins to two. Bins from which contigs were removed during refinement, due to shared contigs among multiple predictions, were tagged with the suffix _sub (Supplementary Table S5).

The quality of the binning process, specifically bin completeness and contamination levels, was further assessed using CheckM v1.2.1 (Parks et al., 2015). CheckM performs phylogenetic placement of bins within a species tree to calculate lineage-specific single-copy marker genes, thereby enabling the evaluation of bin completeness. As detailed in Supplementary Table S5, the CheckM analysis provided comprehensive information on various features of the MAGs. Only one MAG (MetaBAT2Refined-0.2) met the criteria for “medium-quality draft MAGs” according to MIMAG standards (Bowers et al., 2017), exhibiting ≥ 50% completeness and ≤ 10% contamination. Finally, the sequencing depths and relative frequencies of each generated MAG were represented, with the median sequencing depth for each refined bin calculated based on the sequencing depth of its constituent contigs in each sample.

#### 2.4.4 Taxonomic assignment of MAGs

The taxonomic assignment of each MAG was computed using the nf-core/mag-Taxonomic-bin-assignment module, based on the approach of CAT/BAT v5.2.3 (Von Meijenfeldt et al., 2019). This pipeline utilizes Prodigal (Hyatt et al., 2010) for open reading frame (ORF) prediction and DIAMOND (Buchfink et al., 2015) to search the translated ORFs against NCBI’s Nucleotide non-redundant protein database (nr), last updated on 2023-11-20. The assignment of each ORF was determined as the Lowest Common Ancestor (LCA) of all hits falling within a specified range (*r* = 5 hits) of the top hit, with the top-hit bit-score assigned to the classification (*f* = 0.3). CAT then employs a voting method where the bit-scores of all classified ORFs supporting a particular classification are summed. The MAG’s taxonomic assignment is ultimately determined by the classification at the lowest taxonomic level reaching a predefined minimum bit-score. For each MAG, the complete taxonomic assignment, including supporting values up to the lowest supported level, was generated.

#### 2.4.5 Abundance estimations

Using the MAG included in the final refined bin set, Salmon v1.10.3 (Patro et al., 2017) was used to estimate the abundance of each contig across the samples. For quantification, the quasi-mapping approach, which maps sequencing reads to target sequences without requiring full alignments, was applied. This method provides accurate abundance estimates while reducing computational time. Then, these contig-level estimates were used to calculate the average abundance of each MAG across all samples. Resulting raw count estimates were imported into R and normalized using the limma package, expressing the data as Counts Per Million (CPM). This normalization ensures comparability across samples by accurately accounting for variations in library size and sequencing depth, which is essential for the downstream Differential Expression Analysis (DEA) using limma/voom (as detailed in 2.6.2).

#### 2.4.6 Functional annotations

Enzyme Commission (EC) numbers for translated predicted genes were obtained using Prokka v1.14.6 (Seemann, 2014), integrated within the nf-core/mag-Functional_annotation pipeline. Gene Ontology (GO) terms—including GO identifiers, term names, and categories such as biological process, molecular function, and cellular component were derived from comprehensive annotation outputs generated by OmicBox software (Conesa et al., 2005) based on direct homology by BLASTp searches against NCBI non-redundant database. The use of OmicBox for GO annotation provides a more sensitive and complete functional assignment based on sequence homology, complementing domain-based annotations. Kyoto Encyclopedia of Genes and Genomes Ortholog (KO) numbers were assigned using the GhostKOALA annotation server (Kanehisa et al., 2016). This approach was selected due to GhostKOALA’s comprehensive coverage and updated KEGG reference databases, which provide a more complete and accurate mapping of metagenomic sequences to metabolic pathways compared to other KO assignment tools. Protein domains and functional sites were identified with InterProScan version 5.75–106.0 (Blum et al., 2021) against the InterPro database release 93.0. This analysis integrates signatures from multiple member databases including Pfam (v35.0), PANTHER (v18.0), CDD (v3.20), NCBIfam (v11.0), ProSiteProfiles (2023_01), and SMART (v9.0), ensuring a broad and detailed functional characterization. InterProScan domain and site annotations were performed through a custom automated pipeline implemented in a Bash script (Script 2). The pipeline workflow includes: (1) verification and automatic installation of the InterProScan package if absent; (2) execution of InterProScan with selected applications (Pfam, SMART, CDD, TIGRFAM, ProSiteProfiles, PANTHER) enabled, producing GO terms and pathway annotations; (3) optimized parallel processing using a user-specified number of CPU cores; (4) post-processing of tab-separated output files to generate comprehensive annotation tables, including domain presence/absence matrices and protein-associated GO and pathway terms. In the downstream analyses, GO categories derived from OmicBox were preferentially used for functional profiling, while InterProScan annotations provided complementary domain- and site-level functional insights.

All resulting annotation files were saved in the same directory, as the input FASTA file, thus providing a consolidated and clean dataset for subsequent downstream analyses. To assess the coverage and richness of annotations, the number of unique proteins annotated by each method/category was quantified. Furthermore, rarefaction curves were generated using the vegan package (version 2.6–4) in R to evaluate the diversity of unique annotations discovered as a function of the number of sampled proteins. For each annotation method (InterPro databases, EC numbers, KO numbers and GO categories), a community matrix was constructed, where rows represented individual proteins and columns represented unique annotations (e.g., Pfam signatures, EC numbers, or GO terms). The specaccum function with “random” method and 1,000 permutations was employed to calculate the mean richness and 95% confidence intervals, providing insights into the annotation saturation and the efficiency of discovering novel annotations. All plots were generated using ggplot2 (version 3.4.4) and combined into a composite figure using patchwork (version 1.1.3), with individual figures exported as high-resolution TIFF files.

### 2.5 Gene abundance data processing and statistical analysis of metagenome data

Gene abundance data, provided as counts per million (CPM) for each gene was processed and analyzed using R (version 4.5.0) and several specialized packages (Script 3). The raw data, provided in a tab-delimited format, contained columns, among others, for gene identifiers and abundance values for individual samples (e.g., C_1K, LD_1K, PET_1K), and abundance values were extracted into a numeric matrix. A condition factor was defined for statistical grouping. For rarefaction analysis, CPM values were rounded to the nearest integer to approximate count data.

Alpha diversity metrics, including Shannon and Simpson indices, were calculated for each sample using the vegan R package to assess the richness and evenness of the functional gene repertoire on log-transformed CPM data. To evaluate sequencing depth adequacy, rarefaction curves were generated for each sample based on the rounded CPM data, utilizing the minimum total number of counts across all samples as the sampling depth.

The overall structural differences in gene abundance profiles among samples were explored using NMDS and Principal Coordinate Analysis (PCoA). Both ordination techniques were performed on a Bray–Curtis dissimilarity matrix, calculated from the CPM abundance data. For NMDS, a two-dimensional ordination was performed, and the stress value was reported. For PCoA, the percentage of variance explained by the first two principal coordinates was calculated.

To statistically test for significant differences in gene abundance profiles among the experimental conditions, a PERMANOVA analysis was conducted using the Bray–Curtis dissimilarity matrix and 999 permutations. The R2 value from the PERMANOVA analysis was used to quantify the proportion of total variance explained by the “Condition” factor, and a pie chart was generated to visualize the explained versus residual variance. Additionally, an Analysis of Similarities (ANOSIM) was performed. Hierarchical clustering was applied to the samples based on the Bray–Curtis dissimilarity matrix using Ward’s method (ward. D2) for linkage.

For exploratory heatmap visualization, a one-way analysis of variance (ANOVA) was performed for each gene identifier, with abundance (CPM) as the dependent variable and condition as the independent categorical variable. *p*-values were adjusted for multiple comparisons using the False Discovery Rate (FDR) method (Benjamini-Hochberg). A heatmap was generated using pheatmap (Kolde, 2025) to visualize the abundance patterns of ANOVA-significant genes, with genes selected as the top 25 most abundant from the statistically significant subset (adjusted *p* < 0.05). The heatmap was row-scaled (Z-score normalization per gene), and both samples and gene labels were hierarchically clustered.

A Venn diagram was constructed using the VennDiagram R package to assess the overlap of prevalent genes, considering a gene “present” if its sum abundance across all replicates for a condition exceeded 0 CPM.

A gene co-occurrence network was constructed to identify statistically significant positive and negative associations between gene abundances across all samples. Spearman’s rank correlation was used, with edges drawn if the absolute correlation coefficient was greater than or equal to 0.7 and the corresponding adjusted *p*-value (Benjamini-Hochberg) was less than or equal to 0.01. The resulting network was visualized using the igraph R package and a Fruchterman-Reingold layout, with edge color indicating positive or negative correlation and node size reflecting their degree.

Finally, Linear Discriminant Analysis Effect Size (LEfSe) analysis was carried out using the trans_diff function from the microeco R package (version 1.14.0) to identify differentially abundant functional biomarkers, with FDR correction applied to LEfSe *p*-values.

### 2.6 Abundance visualization and enrichment analysis

From all diverse annotation datasets, consolidated into a single, comprehensive dataframe linked by unique protein accessions for downstream analysis, the following analysis were conducted (Script 4).

#### 2.6.1 Abundance visualization and pre-differential expression analysis (pre-DEA)

To gain insights into the overall functional landscape, the accumulated CPMs for selected top 15 annotations (EC numbers, GO terms, KO numbers, and InterPro methods) were visualized across all conditions using boxplots. Separate plots were generated to highlight the top 15 annotations specific to each condition (C, LD, and PET), while still displaying their distribution across all three conditions. This approach enabled the identification of prevalent functional categories within each environmental context and facilitated comparison of their relative abundances.

Furthermore, a Pre-DEA analysis was performed by calculating the log2 fold change (log2FC) of mean CPMs between condition pairs (C vs. LD, C vs. PET, and LD vs. PET). Genes exhibiting a |log2FC| greater than 1 were categorized as over- or under-represented. Functional enrichment analysis (detailed below) was then applied to these sets of genes to identify significantly enriched terms based on fold change alone, prior to rigorous statistical testing.

#### 2.6.2 Differential expression analysis (DEA)

For differential expression analysis (DEA), CPM values were filtered to remove genes with zero counts across all samples. Filtered CPMs were then transformed using the voom method (log2​(CPM + 0.5)) from the limma package to model the mean–variance relationship and stabilize variance. Differential abundance between conditions (C vs. LD, C vs. PET, and LD vs. PET) was determined using the limma R package (Ritchie et al., 2015). Significant differentially abundant proteins were identified based on an adjusted *p*-value (Benjamini-Hochberg) of less than 0.05 and a log_2_ fold-change (log2FC) greater than 0 (over-represented) or less than 0 (under-represented).

#### 2.6.3 Functional enrichment analysis

Functional enrichment analysis was performed using two complementary approaches: Over-Representation Analysis (ORA) and Gene Set Enrichment Analysis (GSEA). ORA was performed on two distinct sets of predicted protein lists:

1. *Pre-DEA enrichment*: Based on the over- and under-represented protein sets derived from the Pre-DEA log_2_FC thresholding. This pre-DEA enrichment analysis aimed to detect functional domains whose aggregated abundance changed across conditions, even when the individual proteins harboring them did not display statistically significant differential abundance—thereby revealing potential functional trends masked at the protein level.
2. *Post-DEA enrichment*: Applied to the sets of significantly over- and under-represented proteins identified from the limma DEA.

In both ORA approaches, functional enrichment was carried out using the clusterProfiler R package (Yu et al., 2012). Gene set size thresholds were set between 5 and 500, and comparisons yielding no eligible gene sets were reported as non-significant. GO term enrichment utilized a custom gene-to-term mapping derived from the consolidated OmicBox annotations. EC number enrichment and InterPro method enrichment similarly used custom gene-to-term mappings. KEGG pathway enrichment was performed using assigned KO numbers. A background set comprising all detected protein accessions was used for each enrichment analysis. For enrichment results, the top 15 terms (ordered by adjusted *p*-value and then by count) were visualized in dot plots. Enriched terms were determined based on a Benjamini-Hochberg adjusted *p*-value of less than 0.05.

GSEA was additionally performed to identify pathways or functional terms that were coherently enriched in a ranked list of all proteins, rather than relying on a hard cut-off of significance as in ORA. This approach is sensitive to subtle but coordinated changes in gene expression across a pathway. For each comparison (C vs. LD, C vs. PET, and LD vs. PET), all detected genes were ranked based on their log_2_ fold change (log_2_FC) of mean CPMs between the two conditions, from the most over-represented to the most under-represented. GSEA was conducted using the fgsea R package (Korotkevich et al., 2019) through the GSEA function within clusterProfiler. Gene set definitions (term-to-gene mappings) for GO terms (Biological Process, Molecular Function, Cellular Component), EC numbers, KO numbers (for KEGG pathways), and InterPro methods were derived from the same comprehensive annotation dataset used for ORA. Gene set size thresholds were set to a minimum of 5 and a maximum of 500. A Benjamini-Hochberg adjusted *p*-value of less than 0.05 was used to determine significant enrichment. The top 15 enriched terms, ordered by adjusted *p*-value and then by Normalized Enrichment Score (NES) were visualized in dot plots.

### 2.7 Sequence homology search and filtering

Predicted protein sequences, derived from the metagenomic assembly MetaBAT2Refined-0.2.faa, were subjected to two separate homology searches. First, they were queried against the NCBI non-redundant (NR) database using BLASTp to obtain general functional annotations at the protein and species level. Second, to specifically identify proteins with homology to enzymes involved in xenobiotic and polymer metabolism, a local BLASTp search was performed against a custom protein database, plasticsdbplus. This custom database was compiled from sequences available in PlasticDB, PAZy, and PMDB. All searches were conducted using NCBI BLAST+ (version 2.16.0+) with multiple CPU cores (−num_threads parameter) for efficient processing. Results were generated in a tabular format (−outfmt 6), and compositional based statistics (comp_based_stats 2) were applied to enhance the accuracy of E-value calculations for potentially distant homologies. Subsequent filtering of the raw BLAST output for the custom database results was performed using custom R scripts (Script 5). Hits were retained based on various criteria, including E-value, percentage identity, and query coverage. Specifically, initial screening criteria required an E-value ≤ 1 × 10^−5^ (reported as *p*-value 0.05 in downstream logs), a minimum sequence identity of 30%, and a query coverage of ≥ 20%. For highly reliable identifications, a stringent secondary threshold of ≥ 50% sequence identity was applied.

The reproducibility of this functional and statistical pipeline is supported by the documented Bash and R scripts (Scripts 2–5) and the standardized software environment detailed in Supplementary Table S6. It should be noted, however, that the statistical sensitivity of the downstream analyses—particularly the differential abundance (limma) and functional enrichment (ORA, GSEA)—is inherently dependent on the initial microbe-to-host DNA ratio. High levels of host DNA contamination can result in a sparse gene abundance matrix, which may limit the detection of statistically significant functional signals even when biological trends are present. This technical constraint should be considered when applying this workflow to host-associated metagenomic datasets with suboptimal microbial recovery.

## 3 Results

To evaluate the impact of plastic-containing diets on the physiology and gut microbiome of *Tenebrio molitor*, larvae were fed for 30 days on wheat bran (WB), WB supplemented with low-density polyethylene (LD), or WB supplemented with polyethylene terephthalate (PET).

### 3.1 Physiological outcomes and survival

All groups exhibited an increase in larval length and weight over time, as shown in Table 1. The survival rates remained high, with 97 and 99% in the LD and PET groups, respectively, indicating no apparent lethal effects of dietary plastic supplementation under the tested conditions.

**Table 1:** Growth performance and survival (SR) rate of *Tenebrio molitor* larvae after dietary exposure to wheat bran (WB), WB supplemented with low-density polyethylene (LD), or WB supplemented with polyethylene terephthalate (PET).

Condition	T0		Tfinal		
	Length (cm)	Weight (mg)	Length (cm)	Weight (mg)	SR (%)
1. Control, 100% wheat bran	1.05 ± 0.03	10.58 ± 0.88	1.27 ± 0.02	16.2 ± 0.47	100%
2. Wheat bran + LD			1.24 ± 0.02	17.6 ± 1.07	97%
3. Wheat bran + PET			1.29 ± 0.03	18.5 ± 1.01	99%

### 3.2 Microbial community and inferred functional shifts (16S rRNA)

**Figure 1:**
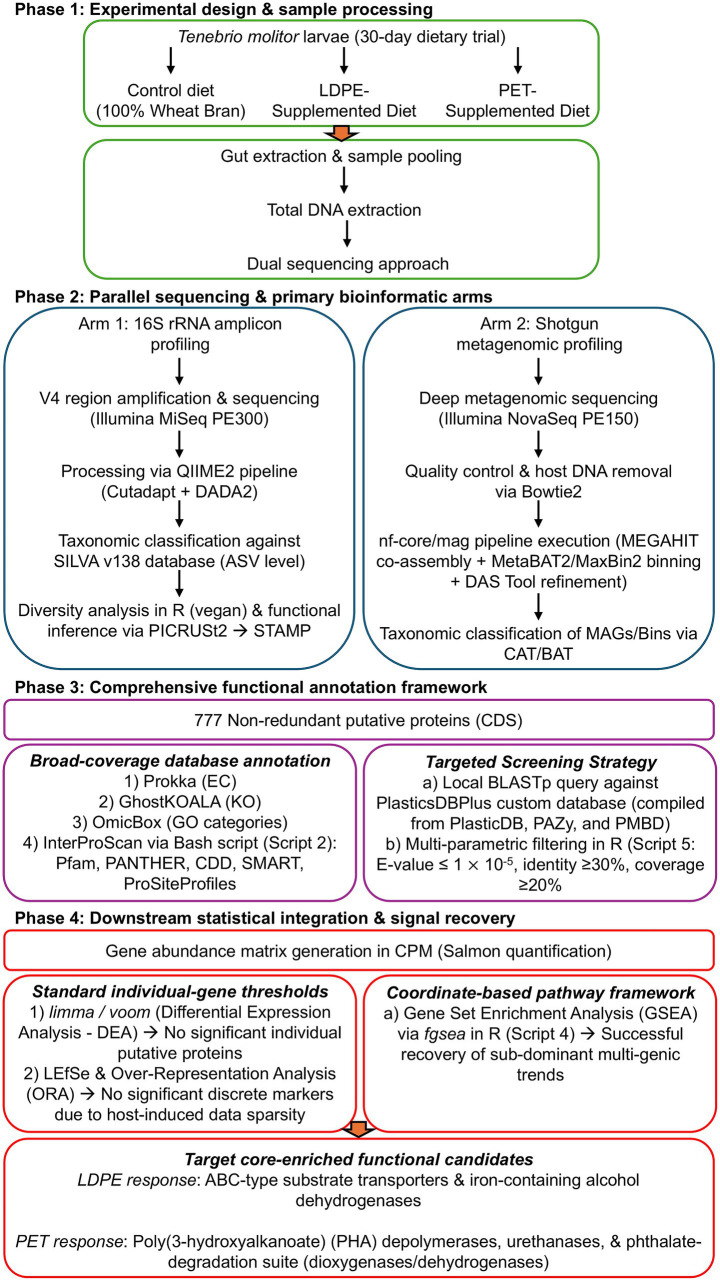
Integrative multi-omic and functional bioinformatic workflow. Schematic representation of the experimental design and data processing pipeline. The framework bifurcates into 16S rRNA amplicon profiling (left arm) for macro-level taxonomic and predicted metabolic assessment, and deep shotgun metagenomics (right arm). Following low-efficiency microbial read recovery due to host DNA filtration, the assembled putative proteome was subjected to parallel broad-coverage database annotation and targeted local BLASTp screening against the custom-curated PlasticsDBPlus repository. Final downstream statistical integration contrasts the failure of standard discrete gene metrics (Limma, LEfSe, ORA) against the robust recovery of coordinated plastic-associated metabolic signals via gene set enrichment analysis (GSEA).

**Figure 2:**
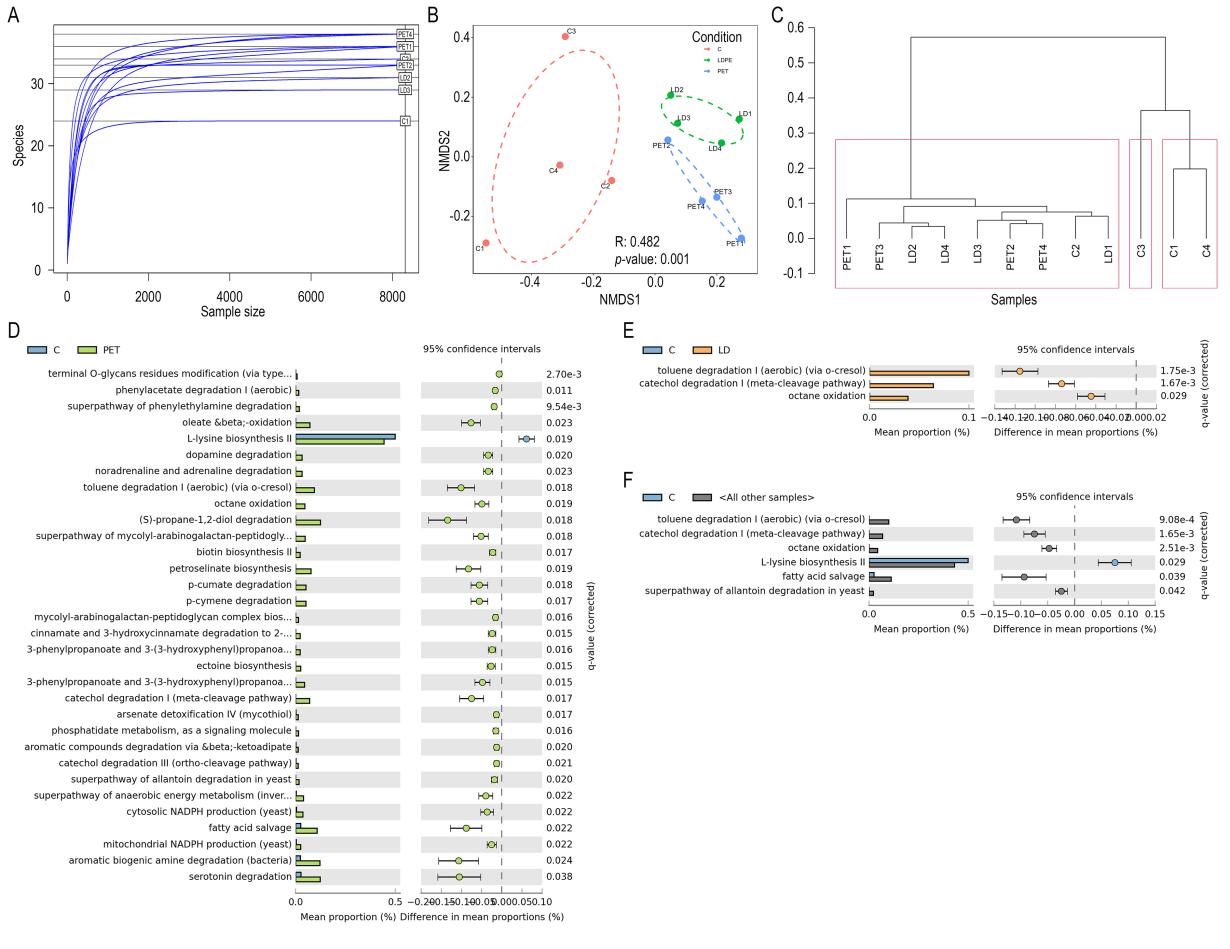
Gut microbiota composition and inferred functional profiles of *Tenebrio molitor* larvae. **(A)** Rarefaction curves of observed ASVs per sample across three dietary groups: control (bran), LDPE, and PET. **(B)** Non-metric multidimensional scaling (NMDS) plot based on Bray–Curtis dissimilarities of ASV-level community composition. **(C)** Hierarchical clustering of samples using Bray–Curtis distances and Ward. D2 linkage method. **(D–F)** Bar plots of predicted functional pathway proportions statistically inferred from 16S rRNA gene amplicon data using PICRUSt2 software and analyzed with STAMP. **(D)** Comparison between PET and control groups, **(E)** comparison between LDPE and control groups, **(F)** comparison between control and all other samples (LDPE + PET). Mean proportions and 95% confidence intervals are shown, along with effect sizes for each pathway. Functional profiles in this figure represent metabolic potential inferred from taxonomic composition, not direct metagenomic sequencing.

To map the comprehensive experimental and computational framework utilized in this study, a detailed multi-omic bioinformatic workflow is integrated and illustrated in Figure 1. *Tenebrio molitor* gut microbiome was influenced by exposure to plastic-supplemented diets (Figure 2). Although all groups approached a plateau in rarefaction curves (Figure 2A), differences in curve elevation indicate variations in ASV richness. These variations were confirmed by a Permutational Multivariate Analysis of Variance (PERMANOVA), which showed significant differences among diet groups (*F* = 4.19, *R^2^* = 0.48, *p* = 0.001). This separation was visualized by the NMDS plot (Figure 2B) and the hierarchical clustering analysis (Figure 2C).

Predicted functional profiles were inferred from the ASV table using PICRUSt2 (Douglas et al., 2020), which generated a table of predicted functional pathway abundances per sample. These functional predictions were statistically analyzed using STAMP (Statistical Analysis of Metagenomic Profiles) software (Parks et al., 2014), which facilitated a visual and statistical comparison of the data (Figures 2D–F). These analyses revealed shifts in the predicted abundance of various metabolic pathways. For instance, in the plastic-fed groups (Panels D and E), altered proportions were observed in pathways related to carbohydrate metabolism, amino acid biosynthesis, and xenobiotic biodegradation and metabolism. Specifically, certain pathways potentially involved in the processing of complex polymers or the utilization of novel carbon sources appeared to be enriched or depleted, such as the metabolism of xenobiotics.

### 3.3 Metagenomic sequencing and assembly quality control

A comprehensive metagenome-wide analysis was meticulously performed to extract all possible information from the available data, aimed to broadly assess the functional potential of the *Tenebrio molitor* gut microbiome across different dietary conditions (C, LD, and PET). Raw sequencing data quality control metrics (Supplementary Figure S1) showed generally good per-base mean quality scores across all samples, predominantly above Q30 for most read positions. A notable observation across all samples was the high proportion of duplicate reads, often exceeding 50% of the total sequence counts (Supplementary Figure S1, “FastQC Sequence Counts” panels). Further analysis of key quality and assembly parameters (Table 2) corroborated these challenges. Despite an excellent depth of raw reads per sample (64–91 million), the percentage of reads retained after quality control (QC) and host removal was remarkably low (6.2–8.3%).

**Table 2:** Summary of metagenomic sequencing and assembly quality metrics.

Domain	Metric	Observed value	Typical/threshold	Diagnosis
Raw data	Raw reads/sample	64–91 million	≥20 million (gut metagenomes)	Excellent depth
	% reads retained after QC & host removal	6.2–8.3%	≥40–65% PF or post-QC reads†	*Very low* → serious loss of usable data
Assembly (MEGAHIT co-assembly)	N50	1,079 bp	>5 kb for well-covered gut datasets	Highly fragmented
	Contigs ≥ 10 kb	6	Hundreds–thousands expected	Insufficient long contigs
	Longest contig	19.5 kb	>50 kb desirable	Short
MAGs	MetaBAT2Refined-0.2	88% complete / 0.28% contamination	HQ ≥ 90% / ≤5%	Near-HQ, acceptable
	MaxBin2Refined-0.002_sub	58% complete/ 20.6% contamination	MQ ≥ 50% / ≤10%	Discard (low quality, high contamination)
Taxonomy	MetaBAT2Refined-0.2	*Enterococcus* (44% support)	Common insect-gut genus	Plausible but low support
	MaxBin2Refined-0.002_sub	*Tenebrio molitor* (host)	Should be microbial	Host contamination

As evidenced by the assembly metrics (Table 2), the co-assembly yielded a highly fragmented output with a low N50 value of 1,079 bp, falling below the expected > 5 kb for well-covered gut metagenomic datasets (Nurk et al., 2017). Furthermore, only 6 contigs were ≥ 10 kb, compared to the hundreds-thousands expected for robust assemblies, and the longest contig was merely 19.5 kb (Table 2), indicating insufficient long contigs.

Metagenome-Assembled Genomes (MAGs) and taxonomic classification (Table 2) also showed challenges. While one MAG (MetaBAT2Refined 0.2) achieved “Medium Quality Draft MAG” status (88% complete / 0.28% contamination), the other (MaxBin2Refined 0.002_sub) was discarded due to high contamination (20.6%). Crucially, taxonomic assignment for MetaBAT2Refined-0.2 MAG showed *Enterococcus* (44% support), while MaxBin2Refined-0.002_sub MAG was identified as host DNA (*Tenebrio molitor*), confirming its necessary exclusion from microbial analyses.

### 3.4 Metagenome-wide functional analysis and gene detection

**Figure 3:**
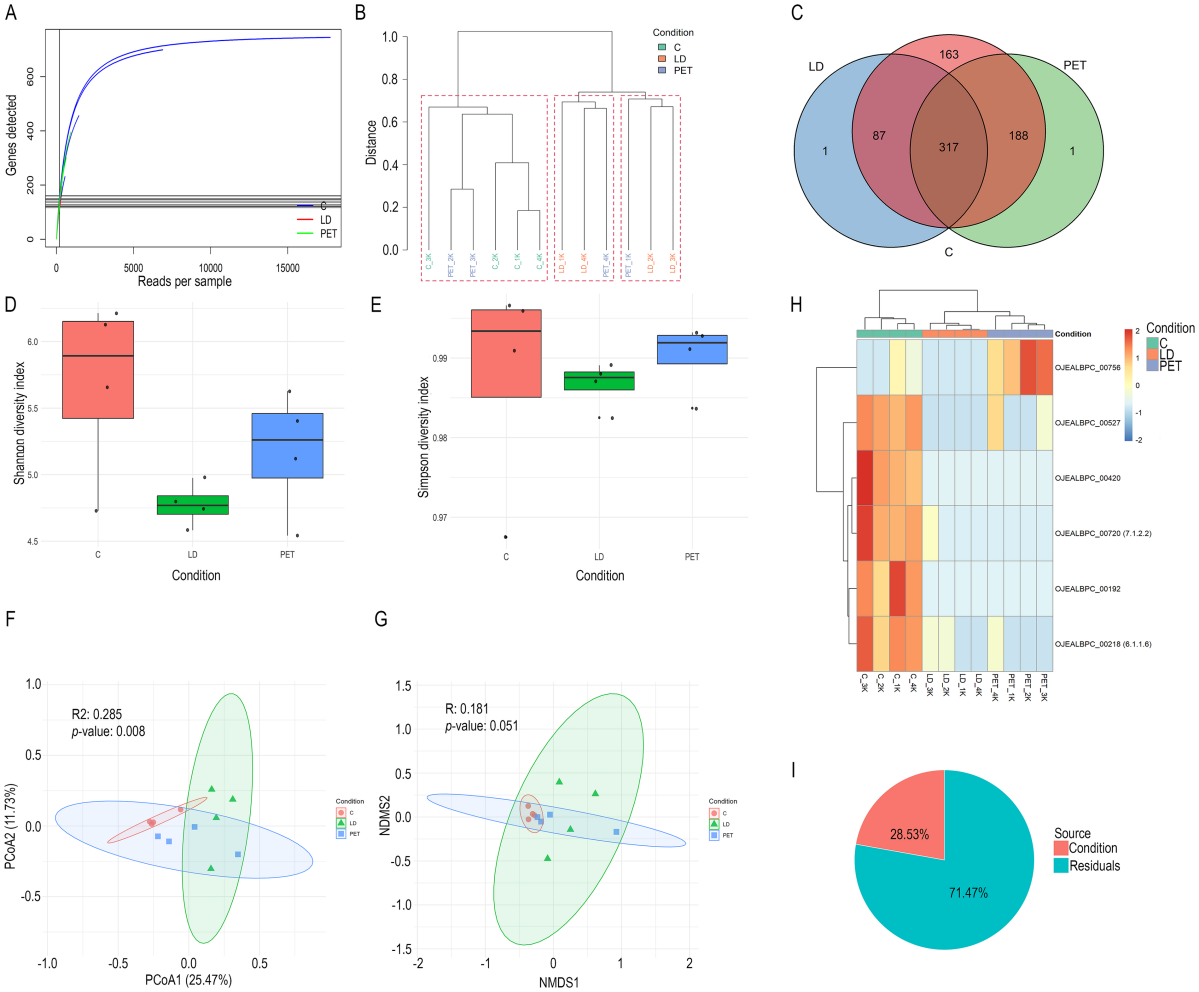
Metagenome-wide functional characterization and community structure based on direct shotgun sequencing. Analysis of gene abundance data derived from shotgun metagenomes of the *Tenebrio molitor* gut, reflecting directly annotated functional profiles under three distinct dietary conditions: control (C), low-density polyethylene (LD), and polyethylene terephthalate (PET) exposure. **(A)** Rarefaction curves by dietary condition. **(B)** Hierarchical clustering dendrogram of samples based on Bray–Curtis dissimilarity, using Ward’s method for linkage. Branches are colored according to experimental condition, and the dendrogram is annotated with three clusters (pink dashed boxes) to highlight correspondence with experimental groups. **(C)** Venn diagram illustrating shared and unique prevalent metagenomic genes across the control, LD, and PET dietary groups. Genes were considered prevalent if their mean abundance exceeded 0.1 CPM within a given condition. **(D,E)** Shannon and Simpson diversity indices, respectively, of the functional gene repertoire. **(F)** Heatmap illustrating the abundance patterns of ANOVA-significant directly annotated genes/proteins. The heatmap displays the top 25 most abundant statistically significant genes (adjusted *p* < 0.05) per condition. Gene abundances are row-scaled (*Z*-score normalized), and both samples and gene labels are hierarchically clustered to reveal co-abundance patterns. The color intensity represents the relative abundance (*Z*-score). **(G)** Principal coordinate analysis (PCoA) plot depicting the linear projection of Bray-Curtis dissimilarities onto orthogonal axes, with explained variance percentages shown for the first two principal coordinates. Samples are colored by condition, with 95% confidence ellipses. **(H)** Non-metric multidimensional scaling (NMDS) ordination plot based on Bray–Curtis dissimilarity, visualizing overall structural differences in metagenomic gene abundance profiles among samples. Samples are colored by condition, with 95% confidence ellipses. The ANOSIM *R*-statistic and *p*-value are indicated. **(I)** Variance partitioning analysis (PERMANOVA) showing the proportion of total variance in shotgun-derived gene profiles explained by the “Condition” factor (*R*^2^ value) versus residual variance.

Functional gene diversity was evaluated. Firstly, it was observed that rarefaction curves of detected genes across dietary conditions failing to reach a plateau for most samples (Figure 3A; Supplementary Figure S3). Likewise, hierarchical clustering based on Bray–Curtis dissimilarity for gene abundance profiles showed mixed compositions (Figure 3B) and Venn diagram showed a 43% of shared genes (Figure 3C). Shannon (Figure 3D) and Simpson (Figure 3E) diversity indices showed variations among the groups.

Ordination plots further elucidated the impact of diet on the overall functional profiles. A PCoA plot (Figure 3F) depicting Bray-Curtis dissimilarities showed some separation of samples by condition along the principal axes. This separation was statistically confirmed by PERMANOVA, which indicated that the ‘Condition’ factor accounted for 28.53% of the total variance (*R^2^* = 0.285, *p* = 0.008), with 95% confidence ellipses. Similarly, the NMDS ordination plot (Figure 3G) also visualized overall structural differences in gene abundance profiles, with an ANOSIM R-statistic of 0.181 and *p*-value of 0.051.

Patterns of gene abundance were also visualized through a heatmap of ANOVA-significant proteins (sequences annotated as CDS) (Figure 3F), displaying the top 25 most abundant statistically significant genes (adjusted-*p* < 0.05). This revealed specific gene clusters that were differentially abundant across the conditions, with specific genes showing upregulation or downregulation in response to C or PET (unclear overrepresentation was found in LD condition). Finally, PERMANOVA revealed that the “Condition” factor accounted for 28.53% of the total variance, while residuals accounted for 71.47% (Figure 3I).

### 3.5 Targeted functional annotation and degradation potential

The protein homology analysis against the NCBI non-redundant database provided a taxonomic perspective on these functional units. From the 777 identified putative proteins (44 of them, 5.7%, described as hypothetical proteins), only 30 unique genera and 44 unique species were resolved through top BLASTP hits (Supplementary Table S7). A striking feature of this taxonomic distribution was the overwhelming dominance of the genus *Enterococcus*, accounting for approximately 87% (677 of 777) of all identified proteins. Specifically, *Enterococcus villorum* (442 proteins) was the most highly represented species. Beyond *Enterococcus*, other genera present in much lower frequencies included *Listeria* (9 proteins), *Streptococcus* (5 proteins), *Bifidobacterium* (3 proteins), *Lactobacillus* (3 proteins), and notably *Microbacterium* (4 proteins), with *Microbacterium tenebrionis* being identified.

Building upon this characterization, a targeted protein homology search was conducted against PlasticDBPlus. Applying initial filtering criteria (*p*-value 0.05; identity 30% and coverage 20%), a total of 14 unique gene-products showed significant homology to entries in PlasticDBPlus. These hits encompassed a range of enzymes directly relevant to xenobiotic and polymer metabolism, including multiple phthalate dioxygenases, poly(3-hydroxybutyrate) (PHB) and poly(3-hydroxyalkanoate) (PHA) depolymerases, polyvinyl alcohol dehydrogenase, urethanase, and 6-aminohexanoate-cyclic-dimer hydrolase (NylA). Further stringent filtering (minimum 50% sequence identity) refined the hits to three unique proteins, all annotated as poly(3-hydroxybutyrate) depolymerases, originating from genera such as *Achromobacter*, *Novosphingobium*, and *Herbaspirillum*.

**Figure 4:**
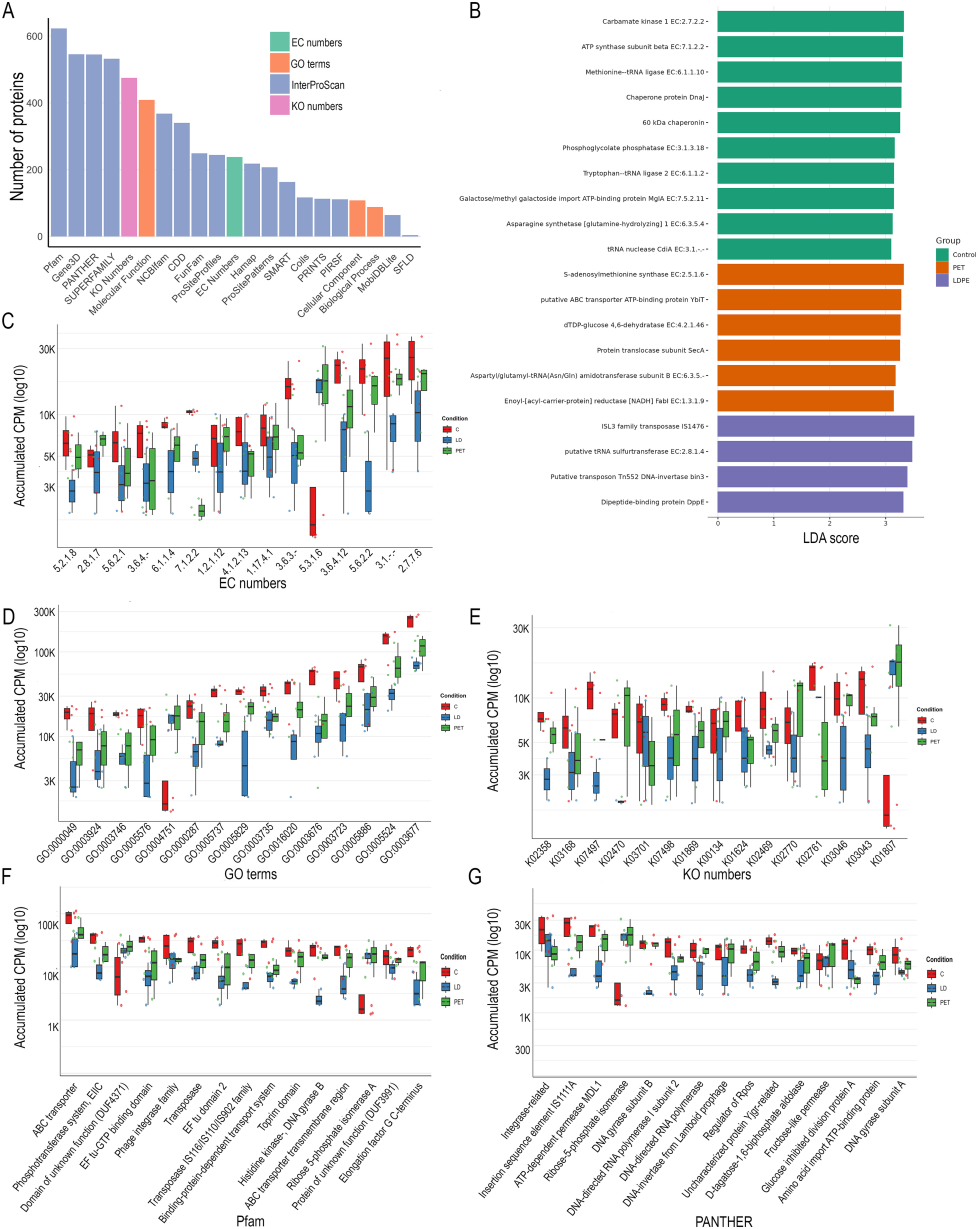
Functional annotation overview and accumulated CPM of directly sequenced metagenomic proteins. **(A)** Number of unique proteins directly annotated from the metagenomic assembly using different functional databases and bioinformatics tools, including Enzyme Commission (EC) numbers, Gene Ontology (GO) terms, InterProScan, and KEGG Orthology (KO) numbers. **(B)** LEfSe (Linear Discriminant Analysis Effect Size) analysis showing the top metagenomic features with the highest LDA scores across experimental conditions (Control, LDPE, PET); note that none were statistically significant. **(C–G)** Accumulated counts per million (CPM) for the top 15 most abundant directly annotated proteins across different functional categories: **(C)** EC numbers, **(D)** GO terms, **(E)** KO numbers, **(F)** Pfam domains, and **(G)** PANTHER protein families. Colors in boxplots indicate experimental conditions (Control (wheat bran) = red, LD (Low-density polyethylene + wheat bran) = blue, PET (polyethylene terephthalate + wheat bran) = green). All functional data in this figure are derived from direct shotgun sequencing reads, representing the observed functional gene repertoire of the larval gut.

Following the initial assessment of metagenomic functional profiles, a comprehensive and rigorous annotation and enrichment analysis was undertaken to maximize insights from the available data (Scripts 3 and 4). This extensive analytical pipeline, including annotation with InterProScan via Bash scripts and subsequent pre-differential expression analysis (Pre-DEA), differential expression analysis (DEA), and post-DEA analyses was carried out in R. These efforts aimed to robustly explore the complex functional potential of the gut microbiome and identify any discernible functional signals related to plastic-associated metabolic pathways, despite the inherent limitations in sequencing depth and assembly quality described previously. The first step involved the annotation of the assembled proteins against various functional databases (Figure 4A). From the total of 777 non-redundant putative proteins identified from the assembled metagenomes, a substantial number of these proteins were successfully annotated. Specifically, 81.1% (630 proteins) with Pfam, 73.3% (570 proteins) with PANTHER, 64.3% (500 proteins) with GO terms, 61.8% (480 proteins) with KEGG Orthology (KO) numbers and 30.6% (238 proteins) with EC numbers.

To gain insights into the overall functional landscape, LEfSe analysis was employed to identify statistically different and biologically relevant genes between the groups, combining non-parametric testing with linear discriminant analysis. However, no significant results were obtained (Figure 4B). Additionally, the accumulated counts per million (CPM) for the top 15 most abundant annotated proteins across various functional categories were visualized across all conditions, in overall and granular forms (Figures 4C–G; Supplementary Figure S2, respectively). This pre-differential expression analysis yielded some visually interesting results. In this regard, ribose-5-phosphate isomerase (EC 5.3.1.6, also identified by CDD RPI_A, Pfam, Superfamily, and KO1807) was consistent among the top 15 overall annotations, with visual indications of over-representation in plastic-exposed conditions. Furthermore, a bacterial ATP-binding cassette transporter from subfamily B (ABCB-BAC; KO6147) was prominent in the top 15 in the LD condition while chitinase (EC 3.2.1.14, also KO1183) appeared in the top 15 in the PET condition.

Given the challenges in identifying statistically significant differential abundance at the individual protein level, GSEA was performed (Supplementary Data Set S1). This approach, which detects subtle but coordinated shifts in entire pathways (Mootha et al., 2003; Subramanian et al., 2005), revealed several significantly enriched functional categories associated with exposure to plastics (PET and LD) compared to control condition. Notably, the ABC transporters family signature (PS00211, InterPro ProSitePatterns) was significantly enriched in the LD-exposed samples. Further strengthening the signal of these functional enrichments, homology analysis against the PlasticDBPlus database identified specific genes within the GSEA core enrichment directly involved in plastic-associated metabolic pathways. These include, among others, poly(3-hydroxyalkanoate) depolymerases genes (e.g., homologous to *Serratia plymuthica* and *Fluoribacter gormanii* enzymes, identified by OJEALBPC_00168 and OJEALBPC_00044), which directly hydrolyze biodegradable polyesters like PHAs (Paloyan et al., 2025), as well as a set of phthalate-related genes (e.g., phthalate 4,5-dioxygenase oxygenase reductase subunit homologous to *Rhodococcus* sp. PBTS 1, by OJEALBPC_00372; 4,5-dihydroxyphthalate decarboxylase homologous to *Loktanella* sp. 22II-4b, by OJEALBPC_00076; phthalate dihydrodiol dehydrogenase homologous to *Gordonia* sp. HS-NH1, by OJEALBPC_00467; and phthalate 4,5-dioxygenase oxygenase subunit homologous to Var*iovorax paradoxus*, by OJEALBPC_00670). The identification of phthalate dihydrodiol dehydrogenase genes, a type of alcohol dehydrogenase, further corroborates the GSEA enrichment of “aldehyde-alcohol dehydrogenases” (FunFam and NCBIfam) and “iron-containing alcohol dehydrogenases” (ProSitePatterns), which were significantly enriched in LD-exposed samples. A polyvinylalcohol dehydrogenase gene (homologous to *Rhodopirellula* sp. SWK7 enzyme, by OJEALBPC_00147), that codes for an enzyme directly involved in the degradation of polyvinyl alcohol (PVA), was also identified and also aligns with the general enrichment of dehydrogenase activities. Finally, a urethanase gene (homologous to *Lysinibacillus fusiformis* enzyme, by OJEALBPC_00521), was also detected.

Complementary to these specific enzymatic roles, fundamental molecular functions such as ATP binding (GO:0005524) and ATP hydrolysis activity (GO:0016887) were broadly enriched in both PET and LD exposure conditions. Domains associated with DNA repair and stress response, such as AAA + ATPases and BRCT domains, and a protein disaggregation chaperone gene product (OJEALBPC_00555) were also enriched.

In an attempt to detect more subtle functional trends, a multi-faceted approach to functional enrichment analysis was employed. Firstly, a Pre-Differential Expression Analysis (Pre-DEA) involved calculating log_2_FC of mean CPMs between condition pairs, categorizing proteins with |log_2_FC| > 1 as over- or under-represented, and then performing enrichment analysis on these sets. This was followed by a more rigorous Post-DEA enrichment, applied to statistically significant differentially abundant proteins identified using the limma R package. However, in both Pre-DEA and Post-DEA functional enrichment analyses for all tested categories, no terms passed the significance threshold based on adjusted *p*-values for any pairwise comparison between conditions (Supplementary Figure S4; Supplementary Data Set S1). In the same direction was the LEfSe analysis (Figure 4B), which did not identify any statistically significant functional biomarkers (adjusted *p* < 0.05) across the experimental conditions.

## 4 Discussion

### 4.1 Physiological resilience and microbial shifts

The high survival rates and sustained growth observed in plastic-fed *T. molitor* larvae (Table 1) are consistent with previous reports demonstrating the tolerance of this organism to plastic-rich diets (Yang et al., 2015; Brandon et al., 2021), and suggest a physiological resilience that warrants further investigation into the underlying microbial mechanisms.

The 16S rRNA analysis confirmed significant shifts in the gut microbiota structure in response to plastic diets (Figure 2), aligning with previous studies demonstrating alterations in *T. molitor* gut microbiota composition in response to various synthetic polymers (Ilijin et al., 2025). Furthermore, the predicted functional changes inferred by PICRUSt2, such as alterations in xenobiotic metabolism, provide valuable, hypothesis-generating insights regarding the metabolic capabilities induced by the plastic diet. Nevertheless, it is important to acknowledge that these functional insights are predictions based on 16S rRNA gene sequences, relying on established databases and phylogenetic inference (Parks et al., 2014). While such predictions offer valuable hypotheses, they do not provide direct evidence of gene presence or expression, a distinction that becomes particularly critical in studies aiming to identify specific plastic-degradation genes. Consequently, these inferred functional profiles should be interpreted with caution, as they represent potential metabolic capacities based on taxonomic correlations rather than the direct detection of the genetic repertoire.

### 4.2 Metagenomic data quality and constraints

The high proportion of duplicate reads (exceeding 50% of the total sequence counts) compromised the ability to resolve specific functional changes. This substantial redundancy, where many reads represented identical sequences, suggested potential issues like limited starting material, over-amplification, or the presence of highly abundant, low-complexity regions (Benjamini and Speed, 2012). While some level of duplication is expected, such high percentages inflate read counts without adding novel sequence information, potentially masking less abundant but functionally important genes by underscoring significant challenges in library preparation or sequencing protocols (MacConaill et al., 2018).

For insect gut metagenomics, studies investigating the functional potential of complex microbial communities often aim for at least 5–10 Gbp of raw data per sample, with an emphasis on unique reads to ensure comprehensive coverage and the ability to detect low-abundance genes (Jin et al., 2022). In broad natural environmental metagenomics, deep sequencing, typically ranging from tens to hundreds of gigabases per sample, is often required to capture comprehensive functional diversity and reconstruct near-complete genomes, especially for less abundant taxa (Cleary et al., 2015). The observed high duplication rates in dataset obtained in this study significantly reduced the effective sequencing depth, likely limiting the detection of such critical functional genes. Further analysis of key quality and assembly parameters (Table 2) corroborated these challenges. Despite an excellent depth of raw reads per sample (64–91 million), surpassing typical recommendations for gut metagenomes (≥ 20 million), the percentage of reads retained after quality control (QC) and host removal was remarkably low (6.2–8.3%). This low retention, significantly below typical thresholds of 40–65% for usable reads, indicates a serious loss of usable data, likely attributable to high duplication and potential host contamination.

As evidenced by the assembly metrics (Table 2), the co-assembly yielded a highly fragmented output with a low N50 value of 1,079 bp, falling below the expected > 5 kb for well-covered gut metagenomic datasets (Nurk et al., 2017). Furthermore, only 6 contigs were ≥ 10 kb, compared to the hundreds-thousands expected for robust assemblies, and the longest contig was merely 19.5 kb (Table 2), indicating insufficient long contigs. This collectively suggest low assembly quality, severely hindering the ability to reconstruct complete genes or genomic regions, which is paramount for accurate functional annotation and gene discovery. This fragmentation severely hindered the ability to reconstruct complete genomic regions or resolve the gene context of specific enzymes. Consequently, the resulting dataset should be interpreted as an exploratory survey of the microbial genetic repertoire rather than a comprehensive metabolic reconstruction (Zhang et al., 2021).

The presence of a Metagenome-Assembled Genome (MAG) classified as *T. molitor* indicates persistent host DNA contamination, even after filtering efforts. Pereira-Marques et al. (2019) highlighted that such high host DNA contamination is a major bottleneck in insect metagenomics, severely limiting microbial signal recovery. Consequently, the highly fragmented co-assembly (low N50 value, few long contigs; Table 2) severely hindered the ability to reconstruct complete genes or operons (Nurk et al., 2017). Also, the high residual variance in the PERMANOVA (71.47%, Figure 3I) further suggests that individual biological variability or technical noise exerted a dominant influence on the observed functional gene profiles, overshadowing the specific effects of plastic diets (Xia, 2023).

To mitigate such issues in future metagenomic studies, optimized experimental strategies are crucial. This includes highly efficient microbial DNA extraction methods to minimize host contamination (Zhou et al., 2014), careful quantification and quality assessment of input DNA, and tailored library preparation protocols that reduce PCR bias or leverage technologies like unique molecular identifiers (UMIs) to distinguish true biological duplicates from PCR artifacts, thereby maximizing the yield of truly unique sequences (MacConaill et al., 2018). Addressing these methodological limitations is essential for obtaining robust metagenomic data capable of revealing the intricate genetic mechanisms underlying plastic degradation in complex microbial communities.

### 4.3 Functional insights: dominance, general metabolism, and putative genetic indicators of plastic metabolism

Since rarefraction curves failed to reach a plateau for most samples (Figure 2A; Supplementary Figure S3), unlike 16S RNA gene data (Figure 2A), could be suggested that the sequencing depth achieved was insufficient to fully capture the complete functional gene repertoire in the plastic-fed groups, implying that some functional diversity remains unexplored after analysis the current data (Zhou et al., 2022). Likewise, hierarchical clustering based on Bray–Curtis dissimilarity for gene abundance profiles further highlighted the complexity and challenges (Figure 3B). This lack of clear diet-specific clustering, contrary to the distinct patterns observed with 16S ASV data (Figure 2C), suggests that the fragmented assembly and incomplete sequencing depth may obscure a clear functional separation among dietary groups, which can also be intuited from the Venn diagram (Figure 3C). Similarly, network analysis of gene co-occurrence, utilizing Spearman’s rank correlation coefficient, revealed a highly interconnected module of genes, suggesting widespread co-expression patterns, albeit without clear sub-modular organization. Indeed, given the non-saturating rarefaction curves, although Shannon (Figure 3D) and Simpson (Figure 3E) diversity indices showed variations among the groups, could be suggested that these metrics may not fully represent the true functional diversity, particularly under the plastic diets where more genes would likely be discovered with deeper sequencing (Zhou et al., 2022). To further assess the impact of this potentially incomplete gene repertoire on community structure, ordination plots such as PCoA and NMDS were then employed to visualize the structural differences in functional gene profiles and identify the extent to which the plastic-diet treatment clustered samples based on Bray–Curtis dissimilarity. While these plots suggest a significant influence of dietary condition, the observed *p*-values, particularly for ANOSIM, are borderline, reflecting the high inter-sample variability and challenges in robustly separating groups based on fragmented metagenomic data (Bharti and Grimm, 2021). Similarly, although significant differences were observed in ANOVA at gene-product level, the overall impact on degradation pathways remains to be conclusively determined due to the limitations in gene detection. Considering also PERMANOVA results, with a high residual variance that outweighs the variance explained by diet, it is strongly suggested that individual biological variability, noise from technical limitations (e.g., incomplete sequencing, host contamination, fragmented assembly), or unmeasured environmental factors exert a dominant influence on the observed functional gene profiles, overshadowing the specific effects of plastic diets (Xia, 2023). Collectively, while these metagenome-wide analyses provide a descriptive overview of the *Tenebrio molitor* gut microbiome’s functional gene content, the substantial limitations in sequencing depth, assembly quality, and the high residual variance restrict definitive conclusions regarding plastic-degradation genes. These findings underscore the critical importance of robust metagenomic data quality for reliable functional inference in complex environmental samples.

The discrepancies between the distinct community clustering observed with 16S ASV data (Figure 2) and the less resolved patterns in metagenomic functional profiles (Figure 3) can be attributed to several factors. First, 16S rRNA gene sequencing provides a robust taxonomic fingerprint, capturing broad shifts in microbial community structure even with relatively shallow sequencing, as ASVs represent operational taxonomic units rather than entire genomes or functional genes (Yen and Johnson, 2021). In contrast, shotgun metagenomics, especially when aiming for functional insights, requires significantly greater sequencing depth to comprehensively detect and assemble full-length genes, particularly those from low-abundance taxa or highly diverse functional repertoires (Parks et al., 2015). The high levels of host contamination and duplicated reads (Table 2) exacerbated this issue, reducing the effective microbial sequencing depth for functional gene discovery (Zhou et al., 2022). Therefore, while microbial community composition might indeed be altered by diet, as shown by ASVs analysis, the functional consequence of these changes might not be fully resolvable without sufficient metagenomic data to capture the underlying genetic pathways. This highlights a crucial methodological point: compositional shifts inferred from marker gene sequencing do not automatically translate to detectable functional shifts in compromised shotgun metagenomic datasets (Jovel et al., 2016).

The protein homology analysis showed a high prevalence of genus *Enterococcus*, which is consistent with previous studies on the *Tenebrio molitor* gut microbiome (Urbanek et al., 2020; Wiegand et al., 2021). While various *Enterococcus* species, particularly *E. faecalis*, have been previously associated with the plastic degradation capabilities of mealworms (Brandon et al., 2021; Yang et al., 2015), it is important to emphasize that its prominence in our dataset does not necessarily imply a unique or specialized role in plastic metabolism. Although other genera were also present in much lower frequencies, some of them associated with plastic degradation in mealworms as *Microbacterium tenebrionis* (Tsochatzis et al., 2021), a strong taxonomic bottleneck is found. The dominance of *Enterococcus* likely reflects its high ecological fitness as a core member of the larval gut microbiota rather than a specific metabolic adaptation to plastic substrates. This dominance further reinforces the notion that the current metagenomic dataset may predominantly reflect the functional contributions of a few highly abundant taxa, rather than the full ecological and functional diversity suggested by marker gene approaches (Jovel et al., 2016). Consequently, conclusions derived from the microbial MAG recovered in this study are specifically representative of the *Enterococcus* lineage rather than the entire community. Nevertheless, the targeted protein homology search using the custom database PlasticsDBPlus, suggested the genomic potential for specific enzymes with putative roles in the degradation of various plastic and synthetic polymers within the *Tenebrio molitor* gut metagenome. However, it is crucial to emphasize that these matches, often characterized by limited sequence identity or coverage, represent putative functional assignments. Therefore, their specific roles in plastic metabolism remain speculative until confirmed by biochemical or transcriptomic evidence. While the absolute number of these proteins with putative plastic-metabolizing functions is small relative to the total predicted proteome, their detection against a specialized database, even with existing metagenomic limitations, suggests that the mealworm gut microbiome harbors genetic features associated with xenobiotic metabolism. This specialized functional capacity, although perhaps not highly abundant or fully resolved in the metagenome, is crucial for understanding the mechanisms underlying plastic biodegradation and complements the broader functional and taxonomic analyses, providing molecular-level indicators of these metabolic capabilities.

According to Yang et al. (2023), it is important to identify specific enzymes in the gut microbiome of plastic-degrading insects to elucidate catabolic pathways, even within a limited dataset, because it provides molecular-level support for a specialized functional capacity. Then, to delve deeper into the possible mechanisms related to the biodegradation of plastics, at the level of putative proteins or domains, functional analysis was carried out. The pipeline let us to robustly explore the complex functional potential of the gut microbiome and identify any discernible functional signals related to plastic-associated metabolic pathways. In this line, it was observed that annotations percentages, particularly for broad-coverage databases like InterProScan and Pfam, are generally consistent with expected rates for identified proteins, where InterProScan often yields higher coverage due to its integration of multiple databases, while more specific annotations like EC or KO numbers typically cover a smaller fraction of the proteome. However, the lower total number of identified proteins inherently limits the breadth of functional insights. In our dataset, the top 15 most abundant annotated putative proteins included enzymes with candidate roles in plastic metabolism, such as the ribose-5-phosphate isomerase, chitinases, and ATP-binding cassette transporters from subfamily B, which were also significantly enriched in the GSEA analysis (Supplementary Data Set S1). Regarding our identification of a key enzyme of the pentose phosphate pathway (ribose-5-phosphate isomerase), its presence could suggest an increased demand for NADPH, critical for reducing oxidative stress and providing precursors for biosynthesis, both essential processes when metabolizing complex substrates or facing environmental challenges (Purohit et al., 2020). Similarly, the over-representation of ABC transporters observed in our samples suggests a potential mechanism where microorganisms could internalize putative breakdown products, such as monomers or oligomers potentially derived from polyethylene, thereby supporting cellular metabolism (Ru et al., 2020), reflecting a generalized transport response to clear hydrophobic fragments from the aliphatic backbone of LDPE. On the other hand, emerging literature indicates the potential of chitinases in plastic biodegradation. For instance, chitinase from certain fungi has been shown to degrade polyethylene (PE) and polyethylene terephthalate (PET) materials. Moreover, some studies comparing PET-degrading enzymes have noted that chitinase or chitinase-like enzymes can exhibit PET-degrading activity, suggesting a more direct role than previously assumed. In the context of our study, this activation alongside the detected aromatic-cleaving pathways could point toward a more specific enzymatic pressure induced by the ester linkages of PET compared to the inert physical stress of LDPE. However, while our analysis identified these enzymes, further research is needed to fully elucidate the extent and mechanisms of their direct involvement in specific plastic degradation pathways, as the well-established primary role of chitinases remains the degradation of chitin-containing biomass in the environment (Ali et al., 2024). These pre-DEA insights provide preliminary indicators of general metabolic upregulation and specific transport mechanisms potentially involved in metabolism of plastic-derived substrates.

In our study, the GSEA analysis, carried out to address the constraints of the fragmented assembly as a robust methodology alternative to full pathway reconstruction, suggested preliminary functional trends in metabolic pathways relevant to plastic-derived substrate processing. To further strengthen these observed functional enrichments, our homology analysis against the PlasticDBPlus database allowed the identification of specific genes. Among them, the identification of poly(3-hydroxyalkanoate) depolymerases can be highlighted, since these enzymes directly hydrolyze biodegradable polyesters like PHAs (Paloyan et al., 2025). Also, a suite of phthalate-related genes (e.g., dioxygenases, dehydrogenases) was detected in our samples; these are known to be critical for breaking down phthalates (Boll et al., 2020; Liu X. et al., 2025). Likewise, a polyvinylalcohol dehydrogenase (Liu et al., 2022) was also identified in our dataset, and it is considered that dehydrogenases are key players in metabolic pathways processing intermediate compounds, such as aldehydes and alcohols, generated during the breakdown of various organic substrates, including those potentially derived from plastic metabolism (Liu S. et al., 2025). Finally, the presence of the urethanase gene in our assembly could suggest a potential capacity for polyurethane degradation, as urethanases cleave urethane linkages found in polyurethanes (Porobic Katnic et al., 2024). Our analysis also indicated a heightened overall metabolic activity and energy demand within the organisms, consistent with the predicted metabolic costs associated with xenobiotic processing, potential nutrient acquisition, and potential stress responses in challenging environments (evidenced in our results by the enrichment in ATP binding and ATP hydrolysis activity functions), adaptation to stress response under exposure conditions (supported by the presence of protein disaggregation chaperone gene) and cellular adaptation to environmental stressors (reflected by the enrichment of domains associated to DNA repair and stress response). Overall, the combined enrichment of transporters and these newly identified candidate enzymes from our dataset suggest a genetic potential for plastic compound processing and metabolism (Mohanan et al., 2020). Beyond the GSEA analysis and the homology analysis, no other significant differences were found (neither in the pre-DEA nor in the post-DEA ORAs). This complete absence of statistically significant functional enrichment, despite extensive efforts, indicates that the limitations in sequencing depth and assembly quality prevented the robust detection of differential functional signals at the individual gene level. Consequently, the functional enrichments identified via GSEA should be interpreted as coordinated metabolic trends rather than discrete, statistically significant changes in individual gene abundances. Consequently, when tracing the implications of each analysis throughout the experimental flow, a clear methodological hierarchy emerges. While 16S rRNA profiling successfully registers macro-level taxonomic responses to plastic ingestion, it lacks the resolution to uncover functional reality. On the other hand, traditional individual-gene differential abundance workflows (such as limma or LEfSe) completely fail under the low-efficiency microbial recovery typical of insect models due to data sparsity. It is only through the combination of coordinate-based gene set frameworks (GSEA) and targeted searches against curated databases (PlasticsDBPlus) that we can extract meaningful genetic indicators from compromised metagenomic datasets. The overarching implication of our workflow is that insect gut microbiomes do possess the genetic potential to sense and process plastic diets, but their functional signals are structurally masked by host genetic backgrounds—meaning future screening efforts must explicitly adapt their statistical strategies to multi-genic trends rather than single-marker tracking.

### 4.4 Methodological and biological limitations of *Tenebrio molitor* as a model for plastic biodegradation

Functional annotations via InterProScan, KO assignment, and custom database comparisons revealed no significant enrichments in pathways related to plastic degradation. ORA of EC numbers, GO terms, and InterPro domains consistently failed to detect enriched gene sets across all contrasts (C vs. LD, C vs. PET, LD vs. PET). While this outcome does not necessarily imply the absence of biodegradative capacity in the gut microbiota of *T. molitor*, it highlights both methodological and biological limitations that restrict interpretation and ecological relevance.

The primary barrier to robust functional inference was technical, stemming from suboptimal metagenomic data quality. Rarefaction analyses indicated undersampling, consistent with current recommendations of ≥10–20 Gb per sample for complex communities (Quince et al., 2017; Treiber et al., 2020). High proportions of host DNA further diluted microbial signal, limiting recovery of complete genes and operons. Short-read assemblies increased fragmentation, while the lack of hybrid or co-assembly strategies likely reduced detection of condition-specific functions. Similar constraints have been reported in other insect microbiome studies, underscoring the importance of host DNA depletion, deeper sequencing, and more flexible gene-prediction pipelines.

From a biological perspective, short-term feeding trials—commonly only a few weeks—may not permit microbial adaptation or selection for plastic-degrading functions. Previous studies suggest that plastic exposure often induces broad stress responses rather than specific catabolic activity. Mamtimin et al. (2023), for instance, showed that larvae fed PS or lignocellulose developed nearly indistinguishable gut microbiomes and transcriptomes, with xenobiotic-like enzyme expression suggesting transient responses.

Homology-based searches against PlasticDBPlus identified candidate enzymes (e.g., alkane hydroxylases, phthalate dioxygenases, urethanases), yet the absence of expression or biochemical validation prevents functional assignment. As emphasized by Zimmermann and Billig (2020) and Li and Zhang (2025), experimental validation is indispensable. Yang et al. (2015) further demonstrated that although ~50% of ingested PS was recovered as CO₂ and frass, <1% was assimilated into biomass, indicating that most plastic remained structurally intact. Similar outcomes have been reported by Lou et al. (2020) and Matyja et al. (2020).

Other limitations include insufficient negative controls, dietary contamination, and the presence of environmental generalists that may confound interpretation (Liu X. et al., 2025; Macan et al., 2025). Regulatory restrictions also shape outcomes: in the European Union, insect farming must use microbiologically safe substrates (Regulation EU 2017/893), this limiting microbial diversity compared with less regulated contexts (Dragone et al., 2025).

Taken together, these constraints underscore the challenges of relying on *T. molitor* as a standalone model for microbial plastic biodegradation. While candidate genes and weak enrichment signals suggest some microbial response to plastic substrates, robust functional shifts remain elusive (Jacquin et al., 2019). Deeper sequencing, longer-term exposures, appropriate controls, and biochemical validation will be essential to clarify whether insect-associated microbiomes contribute meaningfully to plastic bioconversion. Until such evidence accumulates, *T. molitor*-based systems should be interpreted cautiously. Our own experience highlights methodological and biological bottlenecks that are largely shared with other studies in this field; future work should explicitly account for these common limitations when designing experiments and interpreting data.

### 4.5 Conclusions and future directions

The integrative analysis of the *Tenebrio molitor* gut microbiota under plastic-enriched diets performed in this work revealed tentative functional signatures that may be linked to plastic metabolism, including candidate genes with a putative degradative potential (e.g., homology to phthalate dioxygenases, urethanases, and polyhydroxyalkanoate depolymerases). GSEA further suggested possible upregulation of ABC-type substrate transporters and oxidative stress-associated enzymes. These preliminary observations support the notion that insect-associated microbiomes can exhibit some degree of metabolic plasticity when exposed to recalcitrant carbon sources. Nevertheless, the resolution of the dataset presented in this work was constrained by methodological bottlenecks, particularly host DNA contamination, read duplication, and limited sequencing depth, which hindered genome reconstruction and functional annotation. Consequently, this study should be positioned as an exploratory and hypothesis-generating survey rather than a definitive functional characterization. While our findings provide molecular-level indicators of metabolic potential, the absence of transcriptomic or enzymatic validation means that the practical role of these enzymes in plastic bioconversion remains to be experimentally confirmed. As a result, enrichment analyses did not reveal robust or reproducible pathways associated with plastic-related metabolism. Beyond these specific results, the study also contributes a reproducible bioinformatics workflow (publicly available at GitHub), which can inform and accelerate future metagenomic investigations.

Our findings point to the potential of *T. molitor* gut microbiota as a reservoir of biodegradative functions, but also underscore the need for optimized experimental strategies. Future studies should prioritize (i) host DNA depletion during extraction, (ii) increasing sequencing depth and hybrid assembly (short + long reads), (iii) extended feeding trials to promote microbial adaptation, (iv) inclusion of appropriate controls, and (v) biochemical validation of enzyme activities. By highlighting common challenges and providing open resources, this work lays a foundation for more robust exploration of insect-associated microbiomes in plastic bioconversion.

## Data Availability

The datasets presented in this study can be found in online repositories. The names of the repository/repositories and accession number(s) can be found at: https://www.ncbi.nlm.nih.gov/, PRJNA1295778.

## Footnotes

1
www.allgenetics.eu

2
www.ncbi.nlm.nih.gov/sra
