# Supplementary material for: Exploring the Tenebrio molitor gut microbiota response to LDPE and PET: putative genetic indicators and methodological insights
Source: Front Microbiol. 2026 Jun 15;17:1746922. doi: 10.3389/fmicb.2026.1746922 (PMC13311072; doi:10.3389/fmicb.2026.1746922)
Supplement: Supplementary file 1 [file Data_Sheet_1.PDF]

Supplementary Material

1.1 Supplementary Figures

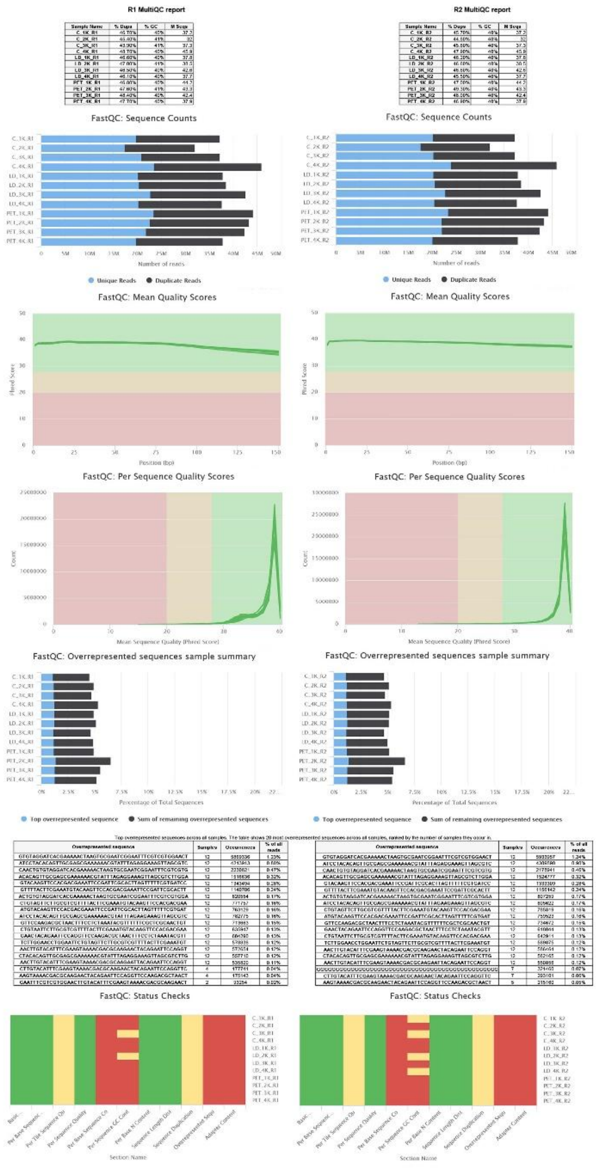

Supplementary Figure 1. Summary of quality control metrics for raw shotgun metagenomic sequencing data. Overview of quality control results for all directly sequenced metagenomic samples, including: (i) MultiQC summary reports for forward (R1) and reverse (R2) reads; (ii) total sequence counts per sample; (iii) FastQC per-base mean quality scores; (iv) FastQC per-base sequence quality scores; (v) FastQC summaries of overrepresented sequences per sample; (vi) the top 20 most overrepresented sequences across all samples; and (vii) FastQC status checks (Pass, Warn, Fail) across various quality parameters. These results confirm overall good sequencing quality of the raw metagenomic reads for downstream analyses.

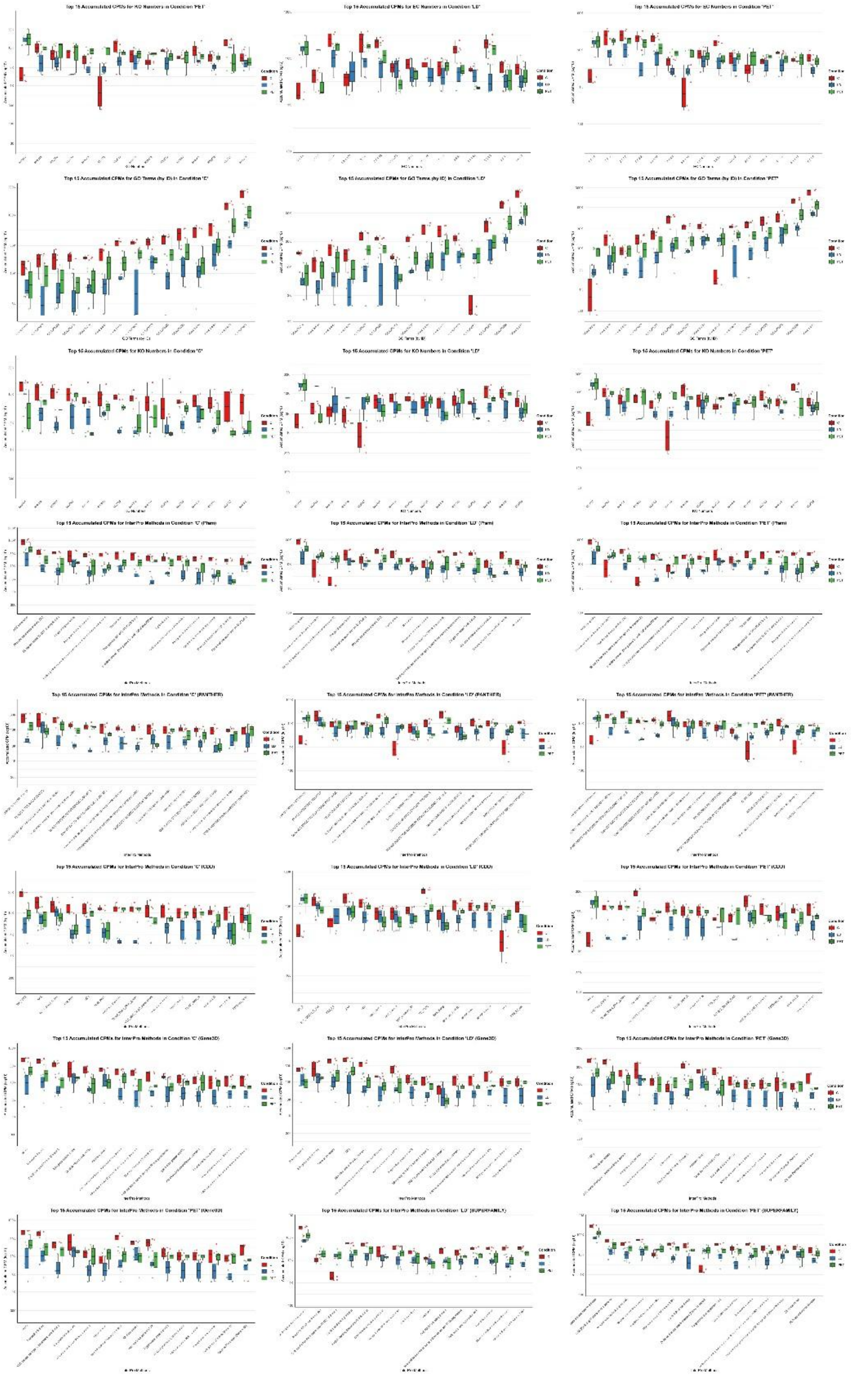

Supplementary Figure 2. Condition-specific top 15 accumulated CPM for directly annotated metagenomic protein functions. Accumulated counts per million (CPM,) of the top 15 most abundant directly annotated proteins from the shotgun metagenomic assembly per condition (Control, LDPE, PET), shown separately for each functional category: Enzyme Commission (EC) numbers, Gene Ontology (GO) terms, KEGG Orthology (KO) numbers, Pfam domains, and PANTHER protein families. Rankings are specific to each condition, highlighting potential shifts in observed functional profiles across treatments derived from direct sequencing reads.

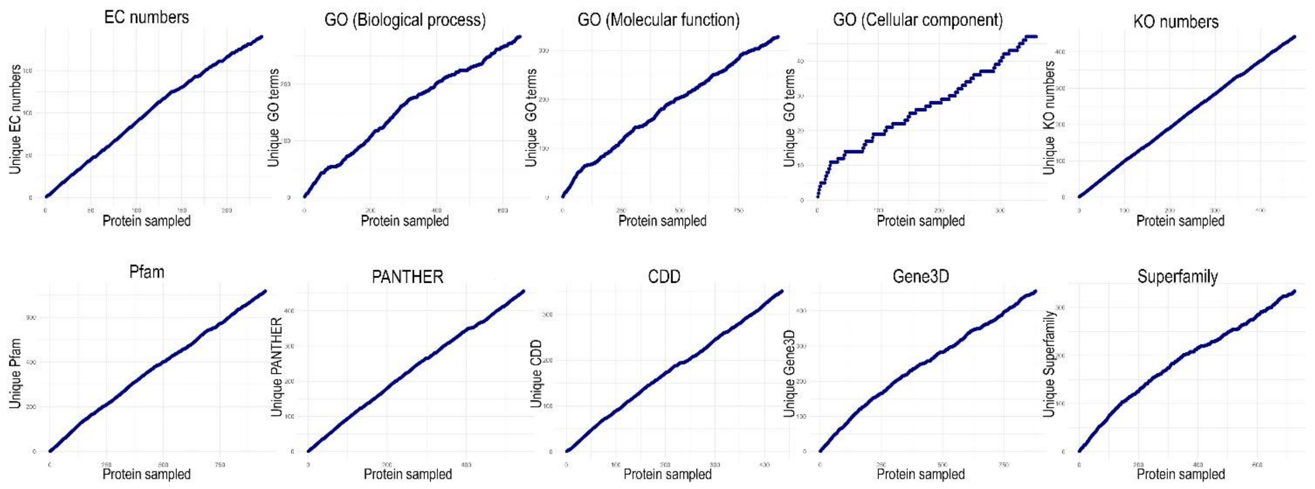

Supplementary Figure 3. Rarefaction curves for directly annotated functional categories. Rarefaction curves showing the accumulation of unique functional annotations as a function of shotgun-derived proteins sampled, based on counts per million (CPM). Curves are shown for ten metagenomic annotation categories: Enzyme Commission (EC) numbers, Gene Ontology (GO) – Biological Process, Molecular Function, and Cellular Component –, KEGG orthology (KO) numbers, Pfam, PANTHER, CDD, Gene3D, and Superfamily. These curves illustrate the diversity and saturation of directly sequenced functional annotations across the assembled protein dataset, confirming adequate depth for functional discovery in the metagenomic reads.

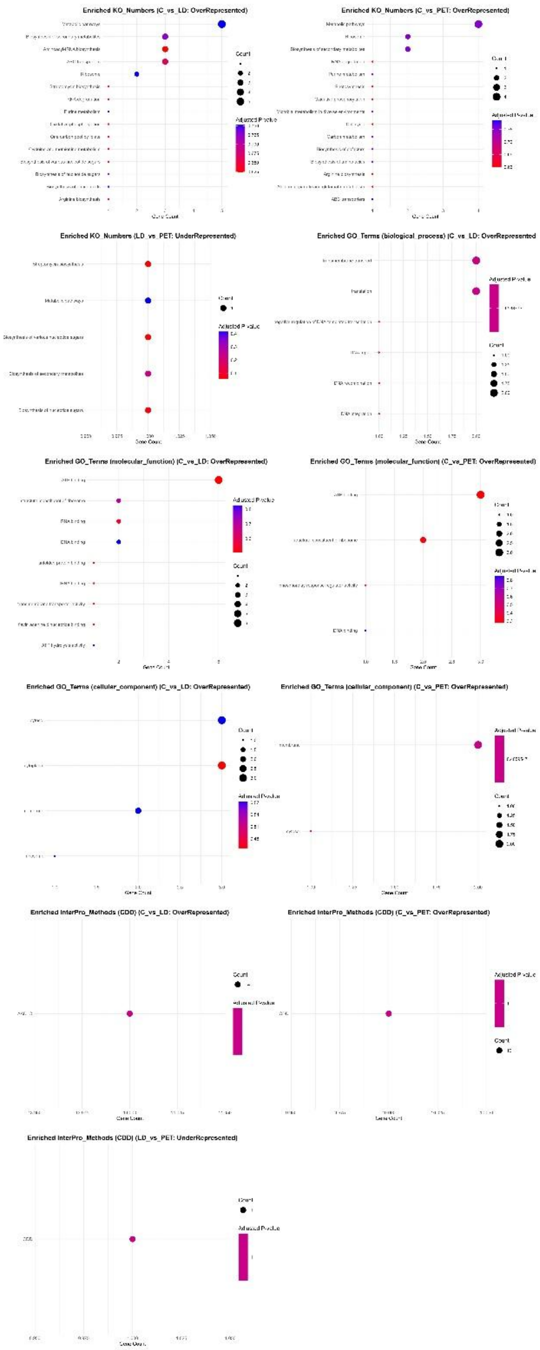

Supplementary Figure 4. Functional enrichment analysis of directly annotated metagenomic genes across experimental conditions. Dot plots summarizing differential enrichment analysis (DEA) results for selected shotgun-derived functional categories: KEGG Orthology (KO) numbers, Gene Ontology (GO) terms (Biological Process, Molecular Function, and Cellular Component), and Conserved Domain Database (CDD). Pairwise comparisons were performed between Control vs. LDPE, Control vs. PET, and LDPE vs. PET. Only categories with observed over- or under-representation are shown; categories or comparisons with empty results (e.g., Pfam, PANTHER, EC numbers) are not included. All functional enrichments displayed were calculated from direct metagenomic protein counts. No terms passed the significance threshold based on Benjamini-Hochberg adjusted *p*-values.

1.2. Supplementary Tables

Table S1. 16S rRNA gene sequencing quality metrics for control and plastic-exposed samples.

Sample ID	Condition	DNA concentration in extracts (ng/µL)	DNA concentration in libraries (ng/µL)	RAW reads (R1+R2)-16S	Paired reads after filtering	ASVs
C1	Control	41.2	18.6	159794	15963	25
C2	Control	47.8	14.3	84730	13658	34
C3	Control	45	10.5	85664	8316	36
C4	Control	47	16.3	111432	13700	38
LD1	LDPE	36.2	4.12	98720	16234	34
LD2	LDPE	42.6	4.66	98354	23533	32
LD3	LDPE	40.4	3.92	84416	16145	29
LD4	LDPE	43.4	5.06	108172	22180	37
PET1	PET	41.8	4.12	95624	25272	36
PET2	PET	44	6.54	83992	19305	33
PET3	PET	41	4.88	108558	25664	38
PET4	PET	40.2	4.38	92714	23696	37
			Total	1212170	223666	

Table 2. Summary of metagenomic sequencing and assembly quality metrics.

Domain	Metric	Observed value	Typical/threshold	Diagnosis
**Raw data**	Raw reads /sample	64–91 million	≥20 million (gut metagenomes)	Excellent depth
	% reads retained after QC & host removal	6.2 – 8.3 %	≥40–65 % PF or post-QC reads†	*Very low* → serious loss of usable data
**Assembly (MEGAHIT co-assembly)**	N50	1,079 bp	>5 kb for well-covered gut datasets	Highly fragmented
	Contigs ≥ 10 kb	6	Hundreds–thousands expected	Insufficient long contigs
	Longest contig	19.5 kb	>50 kb desirable	Short
**MAGs**	MetaBAT2Refined-0.2	88 % complete / 0.28 % contamination	HQ ≥90 % / ≤5 %	Near-HQ, acceptable
	MaxBin2Refined-0.002_sub	58 % complete/ 20.6 % contamination	MQ ≥50 % / ≤10 %	Discard (low quality, high contamination)
**Taxonomy**	MetaBAT2Refined-0.2	*Enterococcus* (44 % support)	Common insect-gut genus	Plausible but low support
	MaxBin2Refined-0.002_sub	*Tenebrio molitor* (host)	Should be microbial	Host contamination

Table S3. Quality metrics of the metagenome co-assembly, computed by QUAST. Statistics are calculated based on scaffolds >= 500 bp.

Assembly metrics	Co-Assembly MEGAHIT
contigs (>= 0 bp)	183731
contigs (>= 1000 bp)	37337
contigs (>= 5000 bp)	164
contigs (>= 10000 bp)	6
contigs (>= 25000 bp)	0
contigs (>= 50000 bp)	0
Total length (>= 0 bp)	137008637
Total length (>= 1000 bp)	58858946
Total length (>= 5000 bp)	1020986
Total length (>= 10000 bp)	74751
Total length (>= 25000 bp)	0
Total length (>= 50000 bp)	0
contigs	106681
Largest contig	19555
Total length	107022081
GC (%)	38.86
N50	1079
N75	738
L50	32188
L75	62318
N's per 100 kbp	0

Table S4. Refined bins parameters.

bin	bin_set	unique_ SCGs	Redundant _SCGs	SCG _set	size	contigs	N50	Bin _score	SCG _completeness	SCG _redundancy
MetaBAT2Refined-0.2	MetaBAT2	45	0	bacteria	880752	315	2768	0.882	88	0
MaxBin2Refined-0.002	MaxBin2	49	27	bacteria	58302466	37058	1560	0.336	96	53
MetaBAT2Refined-0.1	MetaBAT2	9	0	bacteria	258307	123	2011	0.176	18	0
MetaBAT2Refined-0.3	MetaBAT2	4	0	archaea	28274999	13399	2041	0.105	11	0

Table S5. MAG characteristics computed by CheckM.

Bin code	Marker Lineage	Completeness (%)	Contamination (%)	Strain Heterogeneity (%)	Length (bp)	N50 (bp)
MaxBin2Refined-0.002_sub	Bacteria (UID203)	58.25	20.6	2.38	36743	1549
MetaBAT2Refined-0.2	Lactobacillales (UID544)	53.82	0.28	50	315	2768

Table S6. Summary of bioinformatics software, reference databases, and key parameters employed in the 16S rRNA and shotgun metagenomic data processing workflows.

Category	Task	Software / Tool	Version	Database / Reference	Key parameters / criteria
**16S rRNA**	Pre-processing	**Qiime2**	2024.2	-	Cutadapt for adapters; FastQC for quality
	Denoising	**DADA2**	1.26	-	ASV generation
	Taxonomy	**scikit-learn**	0.24.1	SILVA v138 SSU	Pre-trained classifier (515F-806R)
	Diversity & Stats	**R** / **vegan**	4.5.0 / 2.6-6.1	-	Rarefaction to 8,316 reads; PERMANOVA (999 perms)
	Prediction	**PICRUSt2**	2.0	MetaCyc	Hidden-state prediction; STAMP (adj. $p < 0.05$)
**Metagenomics**	Host Removal	**Bowtie2**	2.5.4	*T. molitor* icTenMoli1.1	Unaligned reads retained
	QC & Trimming	**Fastp**	0.23.4	-	Mean quality > 30; Length > 80 bp
	Pipeline Mgr	**Nextflow / mag**	24.10.2 / 3.2.1	-	Integrated workflow
	Taxonomy (Raw)	**Kraken 2**	2.1.3	PlusPFP	Standard *k*-mer classification
	Assembly	**MEGAHIT**	1.2.9	-	*k*-mers: 21,29,39,59,79,99,119; min-count 2
	Binning	**MetaBAT2** / **MaxBin2**	2.15 / 2.2.7	-	Contig threshold: 1500 bp (MetaBAT2) / 1000 bp (MaxBin2)
	Bin Refinement	**DAS Tool**	1.1.6	SCGs	Selection of optimal bins
	Bin Quality	**CheckM**	1.2.1	Lineage-specific	Medium-quality: ≥50% comp, ≤10% cont
	MAG Taxonomy	**CAT/BAT**	5.2.3	NCBI nr (2023-11-20)	Prodigal (ORF); DIAMOND (r=5, f=0.3)
	Abundance	**Salmon**	1.10.3	-	Quasi-mapping; CPM normalization
**Annotation**	General / EC	**Prokka**	1.14.6	-	Integrated in *nf*-core/mag
	GO Terms	**OmicBox**	6.0	NCBI nr	BLASTp-based sensitive assignment
	KO Numbers	**GhostKOALA**	3.1	KEGG	Web-server annotation
	Domains / Sites	**InterProScan**	5.75-106.0	InterPro v93.0	Pfam 35.0, PANTHER 18.0, CDD 3.20

**Statistics**	DEA	**limma / voom**	3.60	-	Adj. $p < 0.05$; log2FC > 0
	Enrichment	**clusterProfiler**	4.12	Custom mappings	ORA & GSEA (Size: 5-500; Adj. $p < 0.05$)
	Homology	**NCBI BLAST+**	2.16.0+	PlasticsDBPlus	local BLASTp; comp_based_stats 2

Table S7. Top hits from Blastp search against custom plastic-degrading enzyme database (plasticsdbplus). This table presents the protein sequences from the *Tenebrio molitor* gut metagenome assembly (derived from MetaBAT2Refined-0.2.faa) that showed significant homology to known plastic and polymer-degrading enzymes present in PlasticDB, PAZy, and PMDB database sequences. Key columns include qseqid (query sequence ID from the metagenome), sseqid (subject sequence ID from plasticsdbplus), stitle (description of the homologous protein, often indicating enzyme type and original organism), pident (percentage sequence identity), evalue (E-value of the hit), and query_coverage (percentage coverage of the query sequence by the hit).

qseqid	sseqid	stitle	pident	length	mismatch	gapopen	qstart	qend	sstart	send	evalue	bitscore	query_length	query_coverage
OJEALBPC_00005	tr|A0A1B5DEL2|A0A1B5DEL2_9PSED	tr|A0A1B5DEL2|A0A1B5DEL2_9PSED Phthalate 4,5-dioxygenase oxygenase reductase subunit OS=Pseudomonas sp. 24 E 1 OX=1844094 GN=pht2 PE=4 SV=1	36.1	36	23	0	1	36	15	50	4.00 E-03	33.5	115	31.3
OJEALBPC_00005	tr|A0A1C3GIW9|A0A1C3GIW9_9PSED	tr|A0A1C3GIW9|A0A1C3GIW9_9PSED Phthalate 4,5-dioxygenase oxygenase reductase subunit OS=Pseudomonas sp. 1 R 17 OX=1844091 GN=pht2_1 PE=4 SV=1	36.1	36	23	0	1	36	15	50	1.00 E-02	32.3	115	31.3
OJEALBPC_00039	tr|Q3MNL0|Q3MNL0_TERSD	tr|Q3MNL0|Q3MNL0_TERSD Transposase subunit OS=Terrabacter sp. (strain DBF63) OX=150395 PE=4 SV=1	31.6	57	38	1	6	61	231	287	6.00 E-03	31.2	66	84.8
OJEALBPC_00044	tr|A0A0W0UBK1|A0A0W0UBK1_9GAMM	tr|A0A0W0UBK1|A0A0W0UBK1_9GAMM Poly(3-hydroxybutyrate) depolymerase OS=Fluoribacter gormanii OX=464 GN=Lgor_0517 PE=4 SV=1	36.2	58	37	0	53	110	196	253	9.00 E-03	34.7	228	25.4
OJEALBPC_00147	tr|M5TC99|M5TC99_9PLAN	tr|M5TC99|M5TC99_9PLAN Polyvinylalcohol dehydrogenase OS=Rhodopirellula sp. SWK7 OX=595460 GN=RRSWK_06183 PE=4 SV=1	30.4	56	29	2	37	92	358	403	5.34 E-04	38.1	202	27.7
OJEALBPC_00168	tr|A0A318P685|A0A318P685_SERPL	tr|A0A318P685|A0A318P685_SERPL Poly(3-hydroxyalkanoate) depolymerase OS=Serratia plymuthica OX=82996 GN=CT690_01845 PE=4 SV=1	35.7	252	152	6	78	324	168	414	7.81 E-35	129	328	75.3
OJEALBPC_00168	tr|K7WJ55|K7WJ55_SERMA	tr|K7WJ55|K7WJ55_SERMA Ketosynthase OS=Serratia marcescens OX=615 GN=oocF PE=3 SV=1	35.7	252	152	6	78	324	168	414	1.94 E-34	128	328	75.3

OJEALBPC_00168	tr|A0A0A8FGB6|A0A0A8FGB6_9GAMM	tr|A0A0A8FGB6|A0A0A8FGB6_9GAMM Poly(3-hydroxyalkanoate) depolymerase OS=Dickeya zeae EC1 OX=1427366 GN=W909_17790 PE=3 SV=1	33.1	287	179	7	47	324	132	414	2.71 E-34	127	328	84.8
OJEALBPC_00168	tr|A0A1Q5WBL1|A0A1Q5WBL1_SERMA	tr|A0A1Q5WBL1|A0A1Q5WBL1_SERMA Poly(3-hydroxyalkanoate) depolymerase OS=Serratia marcescens OX=615 GN=A8A12_04315 PE=3 SV=1	33.9	289	174	8	47	324	132	414	5.82 E-34	127	328	84.8
OJEALBPC_00232	tr|Q3MNL2|Q3MNL2_TERSD	tr|Q3MNL2|Q3MNL2_TERSD Putative regulatory protein OS=Terrabacter sp. (strain DBF63) OX=150395 PE=4 SV=1	37.6	109	59	4	1	106	1	103	1.32 E-21	80.9	113	93.8
OJEALBPC_00380	00218||Chitinase||Geomyces_sp.||PBS_PBSA_PCL	00218||Chitinase||Geomyces_sp.||PBS_PBSA_PCL	36.6	71	43	2	1	70	119	188	2.74 E-09	50.8	105	66.7
OJEALBPC_00458	tr|A0A0N6ZC51|A0A0N6ZC51_9ACTN	tr|A0A0N6ZC51|A0A0N6ZC51_9ACTN Phthalate dioxygenase ferredoxin reductase subunit OS=Gordonia sp. HS-NH1 OX=1435068 PE=4 SV=1	31.0	145	91	5	179	320	1	139	6.28 E-08	51.2	450	31.6
OJEALBPC_00458	tr|A0A285V611|A0A285V611_9ACTN	tr|A0A285V611|A0A285V611_9ACTN Phthalate 3,4-dioxygenase ferredoxin reductase subunit OS=Blastococcus aggregatus OX=38502 GN=SAMN05660748_2226 PE=4 SV=1	30.7	225	132	9	100	313	101	312	6.56 E-05	42.7	450	47.6
OJEALBPC_00458	tr|R4T0C5|R4T0C5_AMYOR	tr|R4T0C5|R4T0C5_AMYOR Phthalate 4,5-dioxygenase reductase subunit OS=Amycolatopsis orientalis HCCB10007 OX=1156913 GN=AORI_1565 PE=4 SV=1	31.5	127	68	6	146	268	108	219	1.00 E-02	35.8	450	27.3
OJEALBPC_00467	tr|A0A0N6ZC47|A0A0N6ZC47_9ACTN	tr|A0A0N6ZC47|A0A0N6ZC47_9ACTN Phthalate dihydrodiol dehydrogenase OS=Gordonia sp. HS-NH1 OX=1435068 PE=4 SV=1	37.1	278	167	3	3	279	11	281	4.91 E-59	187	283	97.9
OJEALBPC_00521	ANG60415.1	ANG60415.1 urethanase [Lysinibacillus fusiformis]	38.5	496	266	6	2	486	3	470	2.23 E-94	292	488	99.4
OJEALBPC_00521	Sporosarcina	Sporosarcina ureae P17a	36.9	491	277	7	3	485	4	469	1.42 E-88	277	488	99.0
OJEALBPC_00521	Agrobacterium	Agrobacterium tumefaciens d3, AmdA	30.9	421	256	8	70	464	90	501	1.10 E-54	189	488	80.9
OJEALBPC_00521	sp|P13397|NYLA_PSES8	sp|P13397|NYLA_PSES8 6-aminohexanoate-cyclic-dimer hydrolase OS=Pseudomonas sp. (strain NK87) OX=314 GN=nylA PE=3 SV=2	31.3	256	159	5	15	262	16	262	9.73 E-20	89.7	488	50.8

OJEALBPC_00521	sp|P13397|NYLA_PSES8	sp|P13397|NYLA_PSES8 6-aminohexanoate-cyclic-dimer hydrolase OS=Pseudomonas sp. (strain NK87) OX=314 GN=nylA PE=3 SV=2	30.4	115	64	4	384	485	373	484	4.71 E-07	50.1	488	20.9
OJEALBPC_00521	sp|P13398|NYLA_PAEUR	sp|P13398|NYLA_PAEUR 6-aminohexanoate-cyclic-dimer hydrolase OS=Paenarthrobacter ureafaciens OX=37931 GN=nylA PE=1 SV=2	31.3	256	159	5	15	262	16	262	2.67 E-19	88.2	488	50.8
OJEALBPC_00521	Metagenome-derived,	Metagenome-derived, UMG-SP-3	32.8	229	148	2	4	226	1	229	1.13 E-14	73.9	488	45.7
OJEALBPC_00521	Metagenome-derived,	Metagenome-derived, UMG-SP-1	35.8	162	99	1	70	226	69	230	1.08 E-10	61.2	488	32.2
OJEALBPC_00555	AEX01748.1	AEX01748.1 protein disaggregation chaperone [Klebsiella michiganensis KCTC 1686]	47.1	746	309	15	66	736	120	854	#######	589	753	89.1
OJEALBPC_00596	tr|A0A2D5WTN4|A0A2D5WTN4_9GAMM	tr|A0A2D5WTN4|A0A2D5WTN4_9GAMM Poly(3-hydroxybutyrate) depolymerase OS=Oceanospirillaceae bacterium OX=1899355 GN=CMI04_10350 PE=4 SV=1	40.0	50	27	2	35	82	323	371	4.00 E-03	32.3	88	54.5
OJEALBPC_00596	tr|A0A176I833|A0A176I833_9GAMM	tr|A0A176I833|A0A176I833_9GAMM Poly(3-hydroxybutyrate) depolymerase OS=Oleibacter sp. HI0075 OX=1822250 GN=A3746_15125 PE=4 SV=1	40.0	50	27	2	35	82	323	371	5.00 E-03	32.3	88	54.5
OJEALBPC_00596	tr|A0A2D6NTC7|A0A2D6NTC7_9GAMM	tr|A0A2D6NTC7|A0A2D6NTC7_9GAMM Poly(3-hydroxybutyrate) depolymerase OS=Oceanospirillaceae bacterium OX=1899355 GN=CMH95_06485 PE=4 SV=1	40.0	50	27	2	35	82	323	371	5.00 E-03	32.3	88	54.5
OJEALBPC_00596	tr|A0A1N7K8U6|A0A1N7K8U6_9GAMM	tr|A0A1N7K8U6|A0A1N7K8U6_9GAMM Poly(3-hydroxybutyrate) depolymerase OS=Oleibacter marinus OX=484498 GN=SAMN05421686_102377 PE=4 SV=1	38.0	50	28	2	35	82	323	371	6.00 E-03	32	88	54.5
OJEALBPC_00663	tr|Q83ZE4|Q83ZE4_TERSD	tr|Q83ZE4|Q83ZE4_TERSD Putative transposase OS=Terrabacter sp. (strain DBF63) OX=150395 GN=ORF20 (ISTesp3) PE=4 SV=1	30.1	133	88	3	1	130	19	149	4.18 E-11	57	142	91.5
OJEALBPC_00670	tr|A0A0H2M766|A0A0H2M766_VARPD	tr|A0A0H2M766|A0A0H2M766_VARPD Phthalate 4,5-dioxygenase oxygenase subunit OS=Variovorax paradoxus OX=34073 GN=pht3 PE=3 SV=1	30.1	475	278	12	10	446	473	931	2.83 E-45	166	446	98.0

1.3. Supplementary Data Set S1.

Supplementary Data Set S1 (SDS1): Pre-DEA, DEA and Post-DEA results (raw and normalized data).
